# High frequency of *HTRA1* AND *ABCC6* mutations in Japanese patients with adult-onset cerebral small vessel disease

**DOI:** 10.1136/jnnp-2022-329917

**Published:** 2022-10-19

**Authors:** Masahiro Uemura, Yuya Hatano, Hiroaki Nozaki, Shoichiro Ando, Hajime Kondo, Akira Hanazono, Akira Iwanaga, Hiroyuki Murota, Yosuke Osakada, Masato Osaki, Masato Kanazawa, Mitsuyasu Kanai, Yoko Shibata, Reiko Saika, Tadashi Miyatake, Hitoshi Aizawa, Takeshi Ikeuchi, Hidekazu Tomimoto, Ikuko Mizuta, Toshiki Mizuno, Tomohiko Ishihara, Osamu Onodera

**Affiliations:** 1 Department of Neurology, Clinical Neuroscience Branch, Brain Research Institute, Niigata University, Niigata, Japan; 2 Department of Medical Technology, Graduate School of Health Sciences, Niigata University, Niigata, Japan; 3 Department of Neurology, Anjo Kosei Hospital, Aichi, Japan; 4 Division of Gastroenterology, Hepato-biliary-pancreatology and Neurology, Akita University, Akita, Japan; 5 Department of Dermatology, Nagasaki University, Nagasaki, Japan; 6 Department of Neurology, Okayama University, Okayama, Japan; 7 Cerebrovascular Medicine, Steel Memorial Yawata Hospital, Fukuoka, Japan; 8 Department of Neurology, National Hospital Organization Takasaki General Medical Center, Gunma, Japan; 9 Department of Neurology, Japanese Red Cross Osaka Hospital, Osaka, Japan; 10 Department of Neurology, Saiki Hospital, Iwate, Japan; 11 Department of Neurology, Tokyo Medical University, Tokyo, Japan; 12 Department of Neurology, Tokyo National Hospital, Tokyo, Japan; 13 Department of Molecular Genetics, Brain Research Institute, Niigata University, Niigata, Japan; 14 Department of Neurology, Mie University, Mie, Japan; 15 Department of Neurology, Graduate School of Medical Science, Kyoto Prefectural University of Medicine, Kyoto, Japan

**Keywords:** VASCULAR DEMENTIA, GENETICS, CEREBROVASCULAR DISEASE, STROKE

## Abstract

**Background:**

This study aimed to clarify the frequency and clinical features of monogenic cerebral small vessel disease (mgCSVD) among patients with adult-onset severe CSVD in Japan.

**Methods:**

This study included patients with adult-onset severe CSVD with an age of onset ≤55 years (group 1) or >55 years and with a positive family history (group 2). After conducting conventional genetic tests for *NOTCH3* and *HTRA1*, whole-exome sequencing was performed on undiagnosed patients. Patients were divided into two groups according to the results of the genetic tests: monogenic and undetermined. The clinical and imaging features were compared between the two groups.

**Results:**

Group 1 and group 2 included 75 and 31 patients, respectively. In total, 30 patients had *NOTCH3* mutations, 11 patients had *HTRA1* mutations, 6 patients had *ABCC6* mutations, 1 patient had a *TREX1* mutation, 1 patient had a *COL4A1* mutation and 1 patient had a *COL4A2* mutation. The total frequency of mutations in *NOTCH3*, *HTRA1* and *ABCC6* was 94.0% in patients with mgCSVD. In group 1, the frequency of a family history of first relatives, hypertension and multiple lacunar infarctions (LIs) differed significantly between the two groups (monogenic vs undetermined; family history of first relatives, 61.0% vs 25.0%, p=0.0015; hypertension, 34.1% vs 63.9%, p=0.0092; multiple LIs, 87.8% vs 63.9%, p=0.0134).

**Conclusions:**

More than 90% of mgCSVDs were diagnosed by screening for *NOTCH3*, *HTRA1* and *ABCC6*. The target sequences for these three genes may efficiently diagnose mgCSVD in Japanese patients.

**WHAT IS ALREADY KNOWN ON THIS TOPIC**

- Monogenic cerebral small vessel disease (mgCSVD) is a major cause of young-onset stroke, dementia and leukoencephalopathy. Cerebral autosomal dominant arteriopathy with subcortical infarcts and leukoencephalopathy is the most common condition. However, the frequency of other mgCSVDs remains unknown.

**WHAT THIS STUDY ADDS**

- Our study revealed that the frequencies of *HTRA1* (20.0%) and *ABCC6* (12.0%) mutations were high among patients with severe CSVD. *NOTCH3*, *HTRA1* or *ABCC6* mutations caused 94% of mgCSVDs.

**HOW THIS STUDY MIGHT AFFECT RESEARCH, PRACTICE OR POLICY**

- Our results showed that screening only three genes can efficiently diagnose mgCSVD in Japan.

## Introduction

Cerebral small vessel disease (CSVD), characterised by lacunar infarction (LI), dilated perivascular spaces (dPVS), microbleeds (MBs) or white matter hyperintensity (WMH) in brain MRI,^1^ causes dementia or gait disturbance (GD).^2^ Although ageing and hypertension (HT) are CSVD risk factors,^3 4^ the pathogenesis of CSVD remains unknown. CSVD is common in the elderly, and most cases are nonfamilial. Currently, more than 10 genes are known to cause monogenic CSVD (mgCSVD) in familial CSVD, including cerebral autosomal dominant arteriopathy with subcortical infarcts and leukoencephalopathy (CADASIL) and high-temperature requirement A serine peptidase 1 (*HTRA*1)-related CSVD.^5 6^ Recently, mgCSVD caused by *HTRA1* mutations has been increasingly reported. It was initially described as a rare recessive disease with characteristic clinical features, but *HTRA1* mutations can cause CSVD, even in heterozygotes. However, the frequency of CSVD caused by *HTRA1* mutations remains unclear.

Diagnosing mgCSVD is challenging in the following respects. First, patients without a family history of CSVD may have mgCSVD. Second, characteristic clinical features are often absent or slight among mgCSVD patients. Therefore, genetic screening is important for diagnosing mgCSVD in patients with CSVD, even in those without a family history. However, it is unclear how and in which cases genetic testing is most effective. In addition, hereditary diseases usually differ among populations. Therefore, it is necessary to optimise the set of genetic tests for each population. Hence, we investigated the frequency and disease spectrum of mgCSVD among adult-onset severe CSVD patients in Japan to answer these questions.

## Material and methods

### Participants

We recruited two groups from patients with adult-onset severe symmetrical WMHs corresponding to Fazekas grade 3/Ⅲ and at least one of the following conditions, including LIs, dPVS, external capsular lesions (ECLs) or MBs^1 7^ on brain MRIs. Group included patients with an age of onset of neurological symptoms/signs, including stroke, GD and/or cognitive impairment (CI)/dementia, ≤55 years irrespective of family history. Group 2 included patients with an age of onset of neurological symptoms/signs, including stroke, GD and/or CI/dementia, >55 and ≤70 years with a family history. Family history was defined as a clear episode of dementia, stroke or leukoencephalopathy in first or second relatives. CI was defined as a score of the Japanese edition Montreal Cognitive Assessment Battery (MoCA-J)<26.^8^

### Genetic tests and measuring HTRA1 protease activity

Genomic DNA was extracted from the blood samples. Conventional genetic tests of exons 2–24 of *NOTCH3* and all the exons of *HTRA1* were performed using a commercially available kit. The CADASIL diagnoses were based on missense mutations with a change in the number of cysteine residues^9^ or previously verified by granular osmiophilic material (GOM) deposition. If retinal vasculopathy with cerebral leukoencephalopathy (RVCL) was suspected according to the clinical or imaging features, a genetic test for exon 2 of *three primer exonuclease 1 (TREX1*) was also performed. The primer set for these three genes has been previously reported elsewhere.^10 11^

If novel mutations in the *HTRA1* gene were identified, we also measured the protease activity of the mutant HTRA1 protein (online supplementals methods and results).

10.1136/jnnp-2022-329917.supp1 Supplementary data

### Whole exome sequencing

We performed whole exome sequencing (WES) on the patients after excluding individuals with *NOTCH3*, *HTRA1* or *TREX1* mutations. Exome analysis was conducted using an outsourcing service (Macrogen, Korea) (online supplementals methods and results), and data analysis of variants was performed using the Macrogen pipeline.

We initially removed synonymous variants, intronic variants or variants with a minor allele frequency of more than 0.01 using the 1000 Genome Phase 3 or Genome Aggregation Database (gnomAD) (https://gnomad.broadinstitute.org/). We then investigated the following mgCSVD-associated genes reported until 2018: *FOXC1, PITX2, COL4A1, COL4A2, CTSA, GLA, CECR1, ABCC6, NF1, CBS, IKBKG, TREX1* and *COLGALT1*.^5 12–14^ In addition, the c-terminus region of *LAMB1* was investigated (online supplemental figure 1).^15^ We evaluated the pathogenicity of the identified mutations using the ClinVar website (https://www.ncbi.nlm.nih.gov/clinvar/) or previous reports. All pathogenic mutations identified using WES were confirmed using conventional Sanger’s methods. The primer set for *ABCC6* has been previously reported.^16^

In addition, we also investigated *COL4A1, COL4A2, ABCC6* and *HTRA1* copy number variants (CNVs) in undiagnosed patients and patients with heterozygous mutations in *ABCC6* and *COL4A2* using copy number estimation by a Mixture of PoissonS (cn.MOPS)^17^ in R software (V.4.1.0). The identified CNVs were verified through a droplet digital PCR (ddPCR) (online supplementals methods and results).

### Classifying the identified mutations

Computational analysis of the identified mutations was performed using PolyPhen2, SIFT and Provean on the VaProS website (http://p4d-info.nig.ac.jp/vapros/). The PHRED score of each mutation’s combined annotation-dependent depletion (https://cadd.gs.washington.edu/) was calculated. The pathogenicity of each mutation was then classified according to the American College of Medical Genetics and Genomics (ACMG) standards and guidelines.^18^

### Clinical and imaging analysis

Clinical information, such as vascular risk factors, neurological symptoms/signs and MoCA-J scores, was collected using a survey sheet. Brain MRI scans of the patients were collected from each centre. The number and locations of the LIs and MBs were investigated. Multiple LIs were defined as more than one.^19^ Multiple MBs were defined as more than four, and the distribution of MBs was classified as strictly lobar or non-lobar.^20^ Severe ECLs were defined as the length of hyperintensity in more than half of the external capsule on T2-weighted images (T2WI)/fluid-attenuated inversion recovery (FLAIR) images.^21^ A severe anterior temporal lesion (ATL) was defined as confluent hyperintensity in the anterior temporal lobe on T2WI/FLAIR images.^21^ LIs, dPVS and MBs were defined according to the Standards for Reporting Vascular Changes on Neuroimaging.^1^

### Statistical analysis

We then divided the included patients into two groups (the mgCSVD and undetermined groups) and statistically compared their clinical and imaging features. Furthermore, we used the genetic test results to divide mgCSVD patients in group 1 into two groups: CADASIL and non-CADASIL mgCSVD. Continuous and categorical variables were compared between the two groups (mgCSVD vs undetermined) using the Wilcoxon rank-sum test and Fisher’s exact test, respectively. Continuous and categorical variables between the three groups (CADASIL vs non-CADASIL vs undetermined) were compared using a one-way analysis of variance and Pearson’s χ^2^ test, respectively. Multiple comparisons using Fisher’s exact tests followed by Hochberg’s correction were performed. We omitted the missing values included in some cases from the statistical analysis. Statistical significance was reached at a p<0.05. Statistical analysis of clinical and imaging features and logistic regression models using stepwise methods to calculate adjusted ORs with 95% CIs were performed using R (V.4.1.2). CSVD patients were classified with the items with no missing values using a decision tree of statistics and a machine learning toolbox on MATLAB R2020b Update 3 (9.9.0.1538559). The optimisation of the hyperparameters in the decision tree was automatically calculated using MATLAB.

## Results

### Identifying CADASIL, cerebral autosomal recessive arteriopathy with subcortical infarcts and leukoencephalopathy (CARASIL) and heterozygous *HTRA1*-related CSVD

We recruited 109 patients from 70 neurological centres throughout Japan (online supplemental table 1). Among them, three patients with leukodystrophy including vanishing white matter disease and X-linked adrenoleukodystrophy were excluded from further analysis. The patients were then divided into group 1 (age of onset at 55 years or younger with or without a family history) and group 2 (age of onset at 55 years or older with family history). Group 1 and group 2 included 75 and 31 patients, respectively (online supplemental table 2). Two patients in group 1 were suspected of having a particular disease based on clinical findings and were diagnosed by genetic testing for those genes. A brain tumour-like episode led us to suspect RVCL and identify a mutation in *TREX1* (p.L287fs).^22^ Another patient was diagnosed with pseudoxanthoma elasticum (PXE) based on skin biopsy and fundus findings, which confirmed a heterozygous compound mutation (p.Q378X and p.L1313fs) in *ABCC6*.

We then performed genetic tests for exons 2–24 of *NOTCH3* and identified 30 patients with CADASIL. All identified mutations had altered numbers of cysteine residues except for p.R75P.^23^ One CADASIL patient had two mutations in *NOTCH3* (p.R607C and p.R1143C). Four mutations (p.C417S, p.G501C, p.Y954C and p.R1371C) have not yet been reported (online supplemental table 3). Clinical features of the four patients with novel *NOTCH3* mutations are summarised in table 1. All four patients had early onset neurological symptoms/signs and extended WMHs on brain MRI, in addition to ECL and LIs. These patients met the diagnostic criteria of CADASIL.^9 24^ Genetic tests for *HTRA1* revealed two patients with CARASIL and nine with heterozygous *HTRA1* mutations.^10 25^ Among patients with heterozygous mutations in *HTRA1*, two mutations (p.L253R and p.V279M) have not yet been reported. The protease activity of both HTRA1 mutants was significantly decreased (online supplementals methods and results and figure 2).

**Table 1:** Clinical features of patients with novel mutations in *NOTCH3*

Mutation	C.1249T>A, p.C417S	C.1501G>T, p.G501C	C.2861A>G, p.Y954C	C.4111C>T, p.R1371C
EGFr domains	10	12	24	34
Sex	Male	Male	Female	Male
Family history*				
First relatives				
Parents	Positive	Positive	Positive	Positive
Children	None	NA	None	None
Second relatives				
Grandparents	NA	NA	None	NA
Sisters/brothers	None	NA	Positive	Positive
Neurological symptoms/signs				
Stroke (years old)	47	None	70	None
CI/Dementia (years old))	47	48	79	52
GD (years old))	None	None	40	None
Migraine	None	None	None	None
Risk factors				
HT	Negative	Positive	Positive	Positive
Smoking	Positive	Negative	Negative	Positive
MRI findings				
WMHs (Fazekas grade)	3 and Ⅲ	3 and Ⅲ	3 and Ⅲ	3 and Ⅲ
LI	Positive	Positive	Positive	Positive
ECL†‡	Moderate	Severe	Severe	Moderate
ATL	Early confluent	Confluent	None	None
Pathological findings				
GOM	NA	Negative	Negative	NA

*Family history is defined as an episode of dementia, stroke or leukoencephalopathy.

†The severity of ECL is classified according to the length of the WMH on the EC.

‡Mild: <1/4 of the EC. Moderate: >1/4 and <1/2 of the EC. Severe: >1/2 of the EC.^21^

ATL, anterior temporal lesion; CI, cognitive impairment; EC, external capsule; ECL, external capsular lesions; EGFr domains, epidermal growth factor-like repeat domains; GD, gait disturbance; GOM, granular osmiophilic material; HT, hypertension; LI, lacunar infarction; NA, not available; WMH, white matter hyperintensity.

### WES and identifying other mgCSVD mutations

We performed WES in the 63 undiagnosed patients. We identified seven patients with mgCSVD. One patient was homozygous (p.M848fs), and another had compound heterozygous mutations (p.W14X and p.M848fs)^16^ in *ABCC6*. The p.W14X mutation is novel. A patient with compound heterozygous *ABCC6* mutations (p.W14X and p.M848fs) was diagnosed with PXE based on xanthoma and angioid streaks. Another patient with a homozygous *ABCC6* mutation (p.M848fs) was lost by follow-up. The other two patients had a heterozygous mutation (p.M848fs) in *ABCC6*. One patient had a mutation in the 3'-untranslated region of *COL4A1* (c.*33T>A), which was previously reported.^26^ Another patient had a novel mutation in *COL4A2* (p.Gly1176del). p.Gly1176 is located in the region of the triplet glycine sequence in *COL4A2*.

We used cn.MOPS^17^ to examine *COL4A1, COL4A2, ABCC6* and *HTRA1* CNVs in 52 undiagnosed patients and three patients with heterozygous mutations in *ABCC6* or *COL4A2*. Binary alignment map file of five undiagnosed patients were unavailable. In one undiagnosed patient, widespread deletion of the region containing the *ABCC6* gene was suspected. ddPCR confirmed the deletion of the *ABCC6* gene at one allele (online supplementals methods and results and figure 3).

Per the ACMG criteria, 14 mutations in *HTRA1*, *ABCC6* and *TREX1* corresponded to pathogenic/likely pathogenic mutations, and 2 mutations in *COL4A1/A2* corresponded to uncertain significance.

Finally, we found that 41/75 patients (54.7%) in group 1 and 9/31 (29.0%) in group 2 had mgCSVD (table 2 and online supplemental table 4). When classified by the presence or absence of a family history, 41.2% of patients in group 1 had mgCSVD, even if there was no family history of the disease. Eight of ten non-CADASIL mgCSVD patients with a family history of first relatives were heterozygous inheritance. Among the mgCSVD types found in group 1, 56.1%, 24.4% and 12.2% of patients had *NOTCH3*, *HTRA1* and *ABCC6* mutations, respectively. In contrast, 22.2% of patients with a family history and 28.6% without a family history had *HTRA1* mutations. Regardless of the presence or absence of a family history, more than 92% of mgCSVD cases could be diagnosed by searching for the three genes.

**Table 2:** Summary of diagnosis

Diagnosis	Group 1 (age of onset of neurological symptoms/signs ≤55 years old)			Group 2 (age of onset of neurological symptoms/signs >55 years old with family history) n=31
	Total n=75	Family history		
		Positive n=41	Negative n=34	
	n (%)	n (%)/(no of patients with a family history of first relatives)	n (%)	n (%)
CADASIL	23 (30.7)	17 (41.5)/(15)	6 (17.6)	7 (22.6)
Heterozygous HTRA1	8 (10.7)	6 (14.6)/(6)	2 (5.9)	1 (3.2)
CARASIL	2 (2.7)	0 (0)/(0)	2 (5.9)	0 (0)
PXE	3 (4.0)	2 (4.9)/(2)	1 (2.9)	0 (0)
Heterozygous ABCC6	2 (2.7)	0 (0)/(0)	2 (5.9)	1 (3.2)
COL4A1	1 (1.3)	0 (0)/(0)	1 (2.9)	0 (0)
COL4A2	1 (1.3)	1 (2.4)(1)	0 (0)	0 (0)
RVCL	1 (1.3)	1 (2.4)(1)	0 (0)	0 (0)
Undetermined	34 (45.3)	14 (34.1)/(9)	20 (58.8)	22 (71.0)

CADASIL, cerebral autosomal dominant arteriopathy with subcortical infarcts and leukoencephalopathy; CARASIL, cerebral autosomal recessive arteriopathy with subcortical infarcts and leukoencephalopathy; COL4A1, COL4A1-related CSVD; COL4A2, COL4A2-related CSVD; Heterozygous ABCC6, heterozygous mutation in ATP Binding Cassette Subfamily C Member 6 (ABCC6); Heterozygous HTRA1, heterozygous high-temperature requirement A serine peptidase 1-related CSVD; PXE, pseudoxanthoma elasticum; RVCL, retinal vasculopathy with cerebral leukoencephalopathy.

In cases with a family history, including those with onset over 55 years of age, 50.0% had mgCSVD. This included 66.7% with CADASIL, 19.4% with *HTRA1* mutations and 8.3% with *ABCC6* mutations.

### Clinical features of mgCSVD patients compared with undetermined patients

We then divided the group 1 patients into two groups (monogenic and undetermined) according to the genetic test or WES results. There were 41 patients with mgCSVD and 34 undetermined patients assigned to each group.

Compared with the undetermined patients, the monogenic group had a significantly higher frequency of a family history of first relatives (61.0% vs 26.5%, p=0.0028), a family history of first and/or second relatives (65.9% vs 41.2%, p=0.0326), positive LIs (92.7% vs 73.5%, p=0.0243), multiple LIs (87.8% vs 67.6%, p=0.0339) and non-lobar MB distributions (22.2% vs 3.4%, p=0.026). The frequency of HT (34.1% vs 64.7%, p=0.0084) was significantly lower in the monogenic group than in the undetermined group (online supplemental table 5). Among these items, a family history of first relatives (OR 1.3325, 95% CI 1.0914 to 1.6268, p=0.0055), HT (OR 0.7408, 95% CI 0.6031 to 0.9098, p=0.0048) and multiple LIs (OR 1.4184, 95% CI 1.1069 to 1.8177, p=0.0064) remained significant in the logistic regression model using stepwise methods (online supplemental methods and results and table 6).

**Figure 1:**
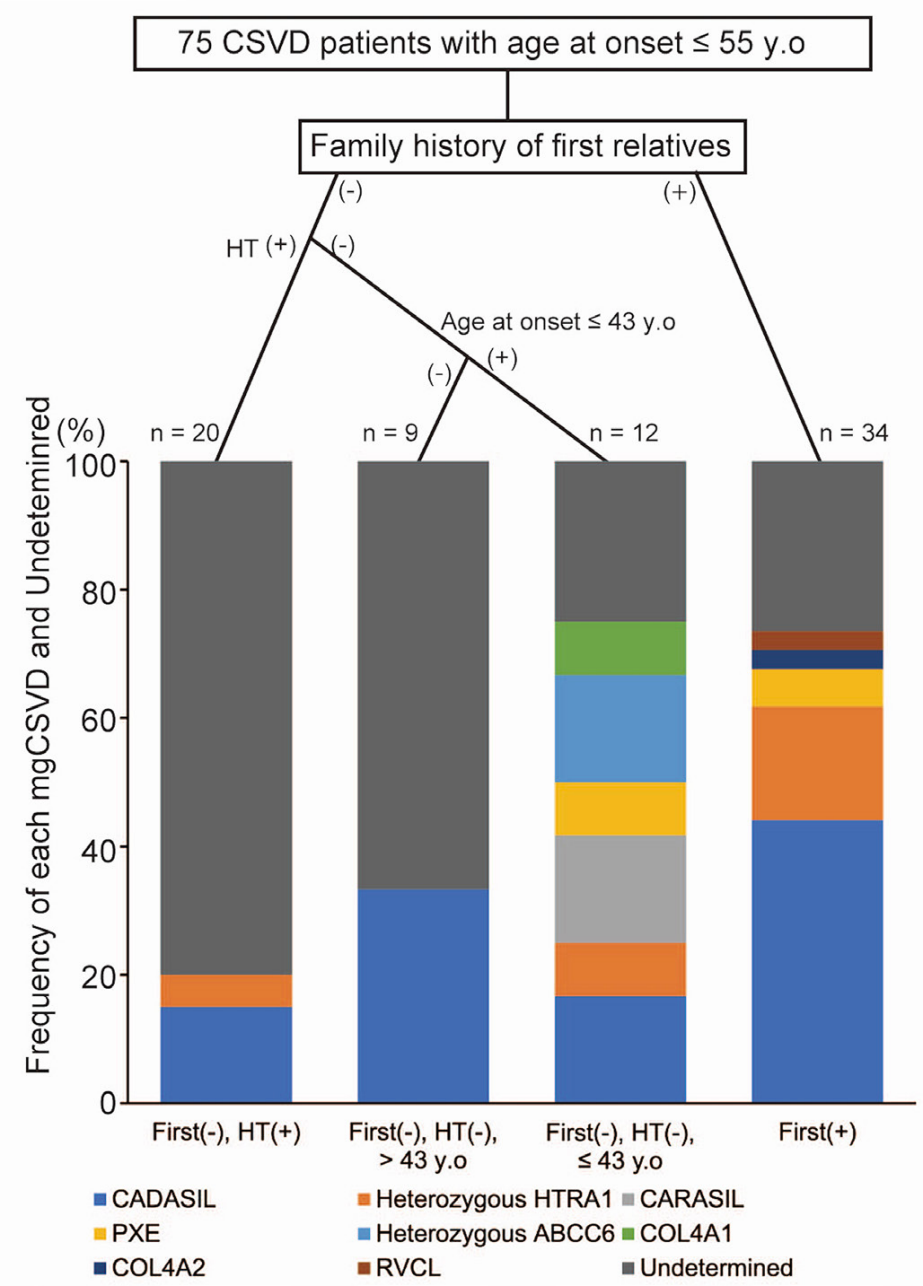
Classifying CSVD patients in group 1 using a decision tree. The bar graph shows the percentage of each mgCSVD and undetermined patient in group 1, classified according to a positive family history of first relatives, HT and the age of onset of neurological symptoms/signs. CADASIL, cerebral autosomal dominant arteriopathy with subcortical infarcts and leukoencephalopathy; CARASIL, cerebral autosomal recessive arteriopathy with subcortical infarcts and leukoencephalopathy; COL4A1, COL4A1-related CSVD; COL4A2, COL4A2-related CSVD; heterozygous ABCC6, heterozygous mutation in ATP binding cassette subfamily C member 6 (ABCC6); HT, hypertension; heterozygous HTRA1, heterozygous high-temperature requirement A serine peptidase 1-related CSVD; PXE, pseudoxanthoma elasticum; RVCL, retinal vasculopathy with cerebral leukoencephalopathy; (-), negative; (+), positive.

Then, we classified the items with no missing values using a decision tree to predict mgCSVD. The results of the decision tree divided group 1 into four groups using three nodes: family history of first relatives, HT and age of onset ≤43 years (figure 1 and online supplemental table 7). In CSVD patients without a family history of first relatives, the frequency of mgCSVD was highest among those without HT and with an age of onset of neurological symptoms/signs ≤43 years (75.0%) and lowest among patients with HT (20.0%). Among CSVD patients with a family history of first relatives, the frequency of mgCSVD was 73.5%. CADASIL was identified in all four groups, and more than four mgCSVD cases were identified in the groups with a positive family history of first relatives and a negative family history, negative HT and an age of onset of neurological symptoms/signs ≤43 yearsd.

Furthermore, we divided the patients into CADASIL and non-CADASIL groups and compared the clinical and imaging features of the three groups (online supplemental table 5). The frequency of a family history of first relatives, HT, GD, alopecia, lumbago/spondylosis deformans, positive LIs, severe ATLs, strict lobar distribution of MBs and non-lobar distribution of MBs differed significantly among the three groups (online supplemental table 5). Multiple comparisons of Fisher’s exact tests followed by Hochberg’s correction showed the following results (table 3). First, the frequency of a family history of first relatives (65.2% vs 26.5%, p=0.0179), LIs (100% vs 73.5%, p=0.0233) and non-lobar distributions of MBs (28.6% vs 3.4%, p=0.0452) in CADASIL patients was significantly higher than in the undetermined group. Second, the frequency of spondylosis deformans or lumbago was significantly higher in the non-CADASIL group than in the CADASIL group (66.7% vs 23.8%, p=0.0316).

**Table 3:** Results of multiple comparison of Fisher’s exact tests followed by Hochberg’s correction between CADASIL, non-CADASIL and undetermined in group 1

	Undetermined versus non-CADASIL	Undetermined versus CADASIL	Non-CADASIL versus CADASIL
Family history			
First relatives	0.1357	0.0179	0.7477
Risk factors			
HT	0.1759	0.0468	0.7417
Neurological symptoms/signs			
GD	0.746	0.0570	0.0570
Extraneurological symptoms/signs			
Alopecia	0.3468	0.1943	0.0632
Spondylosis deformans or lumbago	0.2452	0.1948	0.0316
Neuroimaging findings			
LI	0.5075	0.0233	0.1531
Severe ATL	0.0780	0.7778	0.0752
Strict lobar distribution of MBs	0.3182	1	0.3333
Non lobar distribution of MBs	0.4241	0.0452	0.4241

Statistical analysis was performed using multiple comparison of Fisher’s exact tests, followed by Hochberg’s correction.

ATL, anterior temporal lesion; CADASIL, cerebral autosomal dominant arteriopathy with subcortical infarct and leukoencephalopathy; DL, dyslipidaemia; GD, gait disturbance; HT, hypertension; LI, lacunar infarction; MBs, microbleeds.

Lastly, we divided the patients in group 2 into two groups: monogenic (9 patients) and undetermined (22 patients). Compared with the undetermined group, the frequencies of LIs at the semiovale (88.9% vs 45.5%, p=0.0261) and LIs in the cerebellum (22.2% vs 0%, p=0.0223) were significantly higher in the monogenic group (online supplemental table 8).

## Discussion

In this study, we found that in a group of patients with severe CSVD developed at 55 years of age or younger, more than 50% of the patients, regardless of family history, had a gene mutation responsible for CSVD. Approximately 40% of patients were diagnosed with mgCSVD without a family history. CADASIL accounted for nearly 60% of the patients, followed by *HTRA1*-related CSVD in approximately a quarter of the patients. The third most common group was *ABCC6*-related CSVD, accounting for approximately 10% of cases. These three genes account for more than 90% of the causes of mgCSVD. When compared by family history, the frequency of CADASIL was lower in the group with no family history, but the frequency of patients with *HTRA1-*related or *ABCC6*-related CSVD did not change markedly between the groups with or without a family history. These results indicate that the presence or absence of a family history is not useful for inferring mgCSVD and its type.

The frequency of patients with *HTRA1*-related CSVD was approximately one-third of the CADASIL frequency. However, *HTRA1* loss-of-function mutations were found in one of the 450 apparently normal individuals in the UK.^27^ *HTRA1* mutations have also been described as risk factors for sporadic CSVD.^28^ These findings suggest that the contribution of *HTRA1* to CSVD may be higher than that previously thought, especially in Japan.

In addition, *ABCC6* is the third most commonly mutated gene in patients with severe CVSD. We identified three cases of PXE and three heterozygous patients with *ABCC6* mutations. Compared with the frequency of carriers of *ABCC6* mutations in East Asia (0.76%),^29^ the frequency of heterozygotes found in this study (2.8%) was clearly higher. *ABCC6* causes PXE through biallelic mutations, and symptoms related to CSVD were previously reported in PXE.^30^ Mutations in *ABCC6* are more frequent in ischaemic stroke patients, including CSVD.^31^ Our results indicate that *ABCC6* mutations may be strong risk factor of severe CSVD, even in the heterozygous state. *ABCC6*-related CSVD should be considered a cause of CSVD in Japanese patients.

We summarised recent reports of causative genes of leukoencephalopathy that were determined using next-generation sequencing in table 4.^32–35^ In these studies, *HTRA1* mutations were the second most frequently identified in one of the four studies.^33^ However, *ABCC6* mutations were not identified in these studies, and this type of mutation was observed only in this study. These differences in the frequency of mgCSVD across studies may be due to differences in the included patients or investigated genes.

**Table 4:** Summary of genetic mutations identified among patients with leukoencephalopathy

	This study	Lynch *et al* 32	Chen *et al* 33	Mönkäre *et al* 34	Kunii *et al* 35
Subjects	Severe WMHs with CSVD, n=106	Progressive neurological syndrome with WMHs, n=100	Younger onset of cognitive decline with WMHs, n=45	CI with WMHs, n=35	Leukoencephalopathy, n=60
Total patients with mutation, n (%)	50 (47.2)	21 (21.0)	20 (44.4)	14 (40.0)	12 (20.0)
Patients with CSVD-related gene mutation, n (%)	50 (47.2)	5 (5.0)	19 (42.2)	7 (20.0)	8 (13.3)
NOTCH3	30 (28.3)	4 (4.0)	17 (37.8)	2 (5.7)	7 (11.7)
HTRA1	11 (10.4)	–	2 (4.4)	1 (2.9)	–
ABCC6	6 (5.7)	–	–	–	–
COL4A1	1 (0.9)	–	–	2 (5.7)	–
COL4A2	1 (0.9)	–	–	1 (2.9)	–
TREX1	1 (0.9)	–	–	–	–
CTSA	0 (0)	1 (1.0)	–	–	–
GLA	0 (0)	–	–	–	1 (1.7)
ITM2B	–	–	–	1 (2.9)	–

ABCC6, ATP binding cassette subfamily C member 6; CI, cognitive impairment; CSVD, cerebral small vessel disease; CTSA, cathepsin A; GLA, galactosidase alpha; HTRA1, high-temperature requirement A serine peptidase 1; ITM2B, integral membrane protein 2B; TREX1, three-prime repair exonuclease-1; WMH, white matter hyperintensity.

Next, we examined the clinical mgCSVD features and that the absence of HT, the presence of a family history of first relatives, and multiple LIs may be useful in hypothesising an mgCSVD diagnosis in patients with an age of onset below 55 years (group 1). In addition, we created a decision tree to classify the patients with CSVD into four groups. The mgCSVD frequency was greater than 70% in both groups. The first group had a positive family history of first relatives, and the second group had no family history of first relatives, no HT and an age of onset ≤43 years. The two groups included all cases of mgCSVD caused by mutations in extremely rare genes (*COL4A1, COL4A2* and *TREX1*) observed in this study.

**Figure 2:**
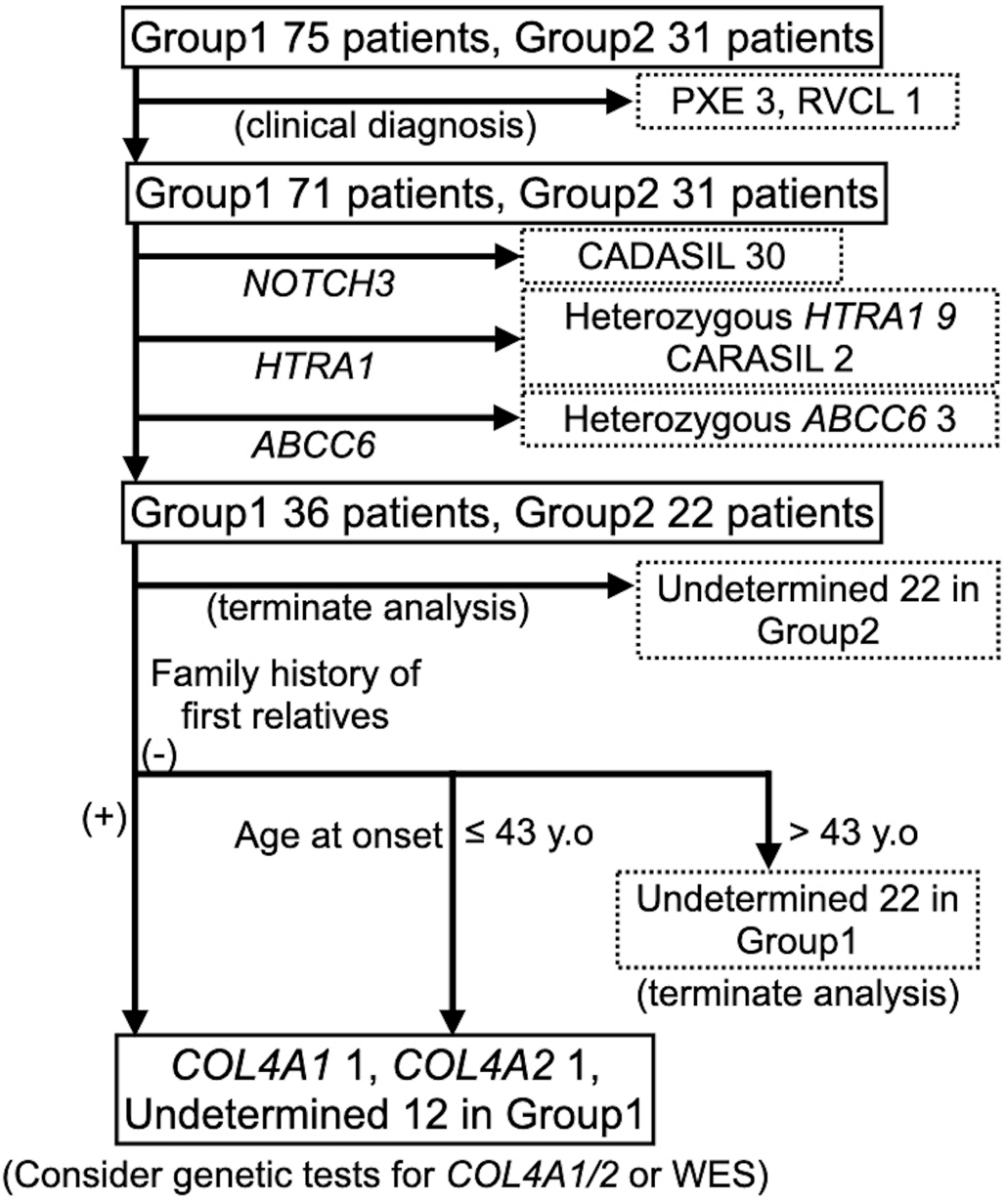
Proposed flow charts for diagnosis of mgCSVD. A proposed flow chart for diagnosing mgCSVD. First, PXE or RVCL are excluded according to clinical and/or imaging features. Second, genetic testing for *NOTCH3* is performed in the remaining patients. Third, *HTRA1* genetic testing is performed in the remaining patients without mutations in *NOTCH3*. Fourth, *ABCC6* genetic testing should be applied to patients without mutations in *NOTCH3* and *HTRA1*. Then, genetic tests should terminate if the patient’s age of onset of neurological symptoms/signs is >55 years. Furthermore, genetic tests should terminate if the patient has an age of onset of neurological symptoms/signs >43 years and a negative family history of first relatives. Additional genetic tests for *COL4A1*, *COL4A2* or WES should be performed for the remaining CSVD patients. CADASIL, cerebral autosomal dominant arteriopathy with subcortical infarcts and leukoencephalopathy; CARASIL, cerebral autosomal recessive arteriopathy with subcortical infarcts and leukoencephalopathy; COL4A1, COL4A1-related CSVD; COL4A2, COL4A2-related CSVD; heterozygous ABCC6, heterozygous mutation in ATP binding cassette subfamily C member 6 (ABCC6); mgCSVD, monogenic cerebral small vessel disease; heterozygous HTRA1, heterozygous high-temperature requirement A serine peptidase 1-related CSVD; PXE, pseudoxanthoma elasticum; RVCL, retinal vasculopathy with cerebral leukoencephalopathy; WES, whole-exome sequencing; (-), negative; (+), positive.

Based on these results, we propose an efficient strategy for genetically testing adult-onset severe CSVD (figure 2). First, PXE or RVCL should be excluded based on clinical or imaging features.^22 36^ Next, we recommend *NOTCH3*, *HTRA1* and *ABCC6* genetic testing. At this step, 96% of mgCSVD cases were diagnosed in the present analysis group. Finally, include *COL4A1/2* genetic testing or WES for patients with an age of onset ≤43 years or an age of onset ≤55 years with a first-degree relative with CSVD. By following this genetic testing strategy, the number of cases requiring WES can be narrowed to 13.2% of the total (14 of 106 patients), using the present analysis as an example.

Our study had some limitations. First, the pathogenicity of several mutations remains unclear. We identified patients with novel *COL4A2* and *NOTCH3* mutations. Mutations in *COL4A2* found in patients with CSVD are usually characterised by the substitution of glycine for another amino acid in the triple repeat sequence.^37 38^ In addition, we did not evaluate the pathogenicity of *COL4A2* mutation such as using skin biopsy.^39^ Hence, it is unclear whether deletion of a single amino acid (glycine) can cause CSVD. While, patients with novel *NOTCH3* mutations met the diagnostic criteria of CADASIL.^9 24^ However, the pathological findings of GOM were negative in two of the four patients with *NOTCH3* mutations. Further studies are required to elucidate the pathogenicity of these mutations. Second, we did not survey the number of relatives with CSVD. Therefore, our study was unable to investigate the association between the number of relatives with or without CSVD and diagnosis of mgCSVD. Thus, our results may be insufficient to clarify the relationship between a diagnosis of mgCSVD and family history. In addition, patients with negative family history included patients for whom information on family history was not available. These points should be clarified in future studies. Third, it is unclear how representative the included patients in this study because there was a bias towards requesting physicians or the requesting institutions were primarily neurology or neurosurgery teaching affiliate institutions. Fourth, we considered that most undetermined patients in this study were caused by vascular risk factors such as HT. Our study indicated that HT was associated with undetermined groups, however, we did not collect detailed information on HT, such as the degree of blood pressure or duration of HT. Therefore, we did not determine how HT contributed to CSVD. In addition, the other possibility is that some of the patients may have genetic mutations that have yet to be elucidated.

## Conclusion

We have shown that *HTRA1* and *ABCC6* mutations are not negligible genetic factors of severe CSVD in Japanese patients. Approximately 40% of the cases were due to mutations in these genes in mgCSVD that developed at 55 years of age or younger. Notably, even heterozygotes can develop severe CSVD. Since these cases are often difficult to diagnose based on clinical features alone, gene testing is necessary. Approximately 90% or more of mgCSVD cases can be diagnosed by screening for these three genes, including *NOTCH3*. All cases are likely to have mutations in these genes because these diseases are widely distributed regardless of family history or age of onset. On the other hand, other rare diseases were identified either in cases with a family history or in cases without a family history or HT and with an age of onset ≤43 years. We believe that targeting this group effectively designates WES as a genetic test for mgCSVD.

## Data Availability

All data relevant to the study are included in the article or uploaded as online supplemental information.

## References

[1] Wardlaw JM , Smith EE , Biessels GJ , et al . Neuroimaging standards for research into small vessel disease and its contribution to ageing and neurodegeneration. Lancet Neurol 2013;12:822–38. 10.1016/S1474-4422(13)70124-8 23867200 PMC3714437

[2] Debette S , Schilling S , Duperron M-G , et al . Clinical significance of magnetic resonance imaging markers of vascular brain injury: a systematic review and meta-analysis. JAMA Neurol 2019;76:81–94. 10.1001/jamaneurol.2018.3122 30422209 PMC6439887

[3] Taylor-Bateman V , Gill D , Georgakis M , et al . Cardiovascular risk factors and MRI markers of cerebral small vessel disease: a mendelian randomization study. Neurology 2021. 10.1212/WNL.0000000000013120. [Epub ahead of print: 29 Nov 2021]. 34845052

[4] Khan U , Porteous L , Hassan A , et al . Risk factor profile of cerebral small vessel disease and its subtypes. J Neurol Neurosurg Psychiatry 2007;78:702–6. 10.1136/jnnp.2006.103549 17210627 PMC2117663

[5] Tan RYY , Markus HS . Monogenic causes of stroke: now and the future. J Neurol 2015;262:2601–16. 10.1007/s00415-015-7794-4 26037017

[6] Uemura M , Nozaki H , Kato T , et al . HTRA1-related cerebral small vessel disease: a review of the literature. Front Neurol 2020;11:545. 10.3389/fneur.2020.00545 32719647 PMC7351529

[7] O'Sullivan M , Jarosz JM , Martin RJ , et al . MRI hyperintensities of the temporal lobe and external capsule in patients with CADASIL. Neurology 2001;56:628–34. 10.1212/WNL.56.5.628 11245715

[8] Ihara M , Okamoto Y , Takahashi R . Suitability of the montreal cognitive assessment versus the mini-mental state examination in detecting vascular cognitive impairment. J Stroke Cerebrovasc Dis 2013;22:737–41. 10.1016/j.jstrokecerebrovasdis.2012.01.001 22306380

[9] Mizuta I , Watanabe-Hosomi A , Koizumi T , et al . New diagnostic criteria for cerebral autosomal dominant arteriopathy with subcortical infarcts and leukocencephalopathy in Japan. J Neurol Sci 2017;381:62–7. 10.1016/j.jns.2017.08.009 28991717

[10] Nozaki H , Kato T , Nihonmatsu M , et al . Distinct molecular mechanisms of HTRA1 mutants in manifesting heterozygotes with CARASIL. Neurology 2016;86:1964–74. 10.1212/WNL.0000000000002694 27164673

[11] Saito R , Nozaki H , Kato T , et al . Retinal vasculopathy with cerebral leukodystrophy: clinicopathologic features of an autopsied patient with a heterozygous TREX 1 mutation. J Neuropathol Exp Neurol 2019;78:181–6. 10.1093/jnen/nly115 30561700

[12] Bugiani M , Kevelam SH , Bakels HS , et al . Cathepsin A-related arteriopathy with strokes and leukoencephalopathy (CARASAL). Neurology 2016;87:1777–86. 10.1212/WNL.0000000000003251 27664989

[13] Müller K , Courtois G , Ursini MV , et al . New insight into the pathogenesis of cerebral small-vessel diseases. Stroke 2017;48:520–7. 10.1161/STROKEAHA.116.012888 28082670

[14] Miyatake S , Schneeberger S , Koyama N , et al . Biallelic COLGALT1 variants are associated with cerebral small vessel disease. Ann Neurol 2018;84:843–53. 10.1002/ana.25367 30412317

[15] Aloui C , Hervé D , Marenne G , et al . End-truncated LAMB1 causes a hippocampal memory defect and a leukoencephalopathy. Ann Neurol 2021;90:962–75. 10.1002/ana.26242 34606115

[16] Iwanaga A , Okubo Y , Yozaki M , et al . Analysis of clinical symptoms and ABCC6 mutations in 76 Japanese patients with pseudoxanthoma elasticum. J Dermatol 2017;44:644–50. 10.1111/1346-8138.13727 28186352

[17] Klambauer G , Schwarzbauer K , Mayr A , et al . cn.MOPS: mixture of Poissons for discovering copy number variations in next-generation sequencing data with a low false discovery rate. Nucleic Acids Res 2012;40:e69. 10.1093/nar/gks003 22302147 PMC3351174

[18] Richards S , Aziz N , Bale S , et al . Standards and guidelines for the interpretation of sequence variants: a joint consensus recommendation of the American College of medical genetics and genomics and the association for molecular pathology. Genet Med 2015;17:405–24. 10.1038/gim.2015.30 25741868 PMC4544753

[19] Arauz A , Murillo L , Cantú C , et al . Prospective study of single and multiple lacunar infarcts using magnetic resonance imaging: risk factors, recurrence, and outcome in 175 consecutive cases. Stroke 2003;34:2453–8. 10.1161/01.STR.0000090351.41662.91 14500936

[20] Zhang J , Liu L , Sun H , et al . Cerebral microbleeds are associated with mild cognitive impairment in patients with hypertension. J Am Heart Assoc 2018;7. 10.1161/JAHA.117.008453. [Epub ahead of print: 01 06 2018]. PMC6015349 29858365

[21] Tomimoto H , Ohtani R , Wakita H , et al . Small artery dementia in Japan: radiological differences between CADASIL, leukoaraiosis and binswanger's disease. Dement Geriatr Cogn Disord 2006;21:162–9. 10.1159/000090677 16391479

[22] Stam AH , Kothari PH , Shaikh A , et al . Retinal vasculopathy with cerebral leukoencephalopathy and systemic manifestations. Brain 2016;139:2909–22. 10.1093/brain/aww217 27604306 PMC5091044

[23] Mizuno T , Muranishi M , Torugun T , et al . Two Japanese CADASIL families exhibiting notch3 mutation R75P not involving cysteine residue. Intern Med 2008;47:2067–72. 10.2169/internalmedicine.47.1391 19043263

[24] Mancuso M , Arnold M , Bersano A , et al . Monogenic cerebral small-vessel diseases: diagnosis and therapy. consensus recommendations of the European academy of neurology. Eur J Neurol 2020;27:909–27. 10.1111/ene.14183 32196841

[25] Yamashita T , Nozaki H , Wakutani Y . A Japanese family of autosomal dominant cerebral small vessel disease with heterozygous HTRA1 mutation showing dementia, gait distrubance and subarachnoid hemorrahge. Vas-Cog Journal 2019;5:20–3.

[26] Sakai N , Uemura M , Kato T , et al . Hemorrhagic cerebral small vessel disease caused by a novel mutation in 3' UTR of collagen type IV alpha 1. Neurol Genet 2020;6:e383. 10.1212/NXG.0000000000000383 32042912 PMC6940479

[27] Malik R , Beaufort N , Frerich S , et al . Whole-exome sequencing reveals a role of HtrA1 and EGFL8 in brain white matter hyperintensities. Brain 2021;144:2670–82. 10.1093/brain/awab253 34626176 PMC8557338

[28] Traylor M , Persyn E , Tomppo L , et al . Genetic basis of lacunar stroke: a pooled analysis of individual patient data and genome-wide association studies. Lancet Neurol 2021;20:351–61. 10.1016/S1474-4422(21)00031-4 33773637 PMC8062914

[29] Grami N , Chong M , Lali R , et al . Global assessment of mendelian stroke genetic prevalence in 101 635 individuals from 7 ethnic groups. Stroke 2020;51:1290–3. 10.1161/STROKEAHA.119.028840 32106772

[30] Pavlovic AM , Zidverc-Trajkovic J , Milovic MM , et al . Cerebral small vessel disease in pseudoxanthoma elasticum: three cases. Can J Neurol Sci 2005;32:115–8. 10.1017/S0317167100016991 15825558

[31] De Vilder EYG , Cardoen S , Hosen MJ , et al . Pathogenic variants in the ABCC6 gene are associated with an increased risk for ischemic stroke. Brain Pathol 2018;28:822–31. 10.1111/bpa.12620 29722917 PMC6581194

[32] Lynch DS , Rodrigues Brandão de Paiva A , Zhang WJ , et al . Clinical and genetic characterization of leukoencephalopathies in adults. Brain 2017;140:1204–11. 10.1093/brain/awx045 28334938 PMC5405235

[33] Chen Z , Tan YJ , Lian MM , et al . High diagnostic utility incorporating a targeted neurodegeneration gene panel with MRI brain diagnostic algorithms in patients with young-onset cognitive impairment with leukodystrophy. Front Neurol 2021;12:631407. 10.3389/fneur.2021.631407 33597917 PMC7882677

[34] Mönkäre S , Kuuluvainen L , Kun-Rodrigues C , et al . Whole-exome sequencing of finnish patients with vascular cognitive impairment. Eur J Hum Genet 2021;29:663–71. 10.1038/s41431-020-00775-9 33268848 PMC8115269

[35] Kunii M , Doi H , Ishii Y , et al . Genetic analysis of adult leukoencephalopathy patients using a custom-designed gene panel. Clin Genet 2018;94:232–8. 10.1111/cge.13371 29700822

[36] Germain DP . Pseudoxanthoma elasticum. Orphanet J Rare Dis 2017;12:85. 10.1186/s13023-017-0639-8 28486967 PMC5424392

[37] Kuo DS , Labelle-Dumais C , Gould DB . COL4A1 and COL4A2 mutations and disease: insights into pathogenic mechanisms and potential therapeutic targets. Hum Mol Genet 2012;21:R97–110. 10.1093/hmg/dds346 22914737 PMC3459649

[38] Zagaglia S , Selch C , Nisevic JR , et al . Neurologic phenotypes associated with COL4A1/2 mutations: expanding the spectrum of disease. Neurology 2018;91:e2078–88. 10.1212/WNL.0000000000006567 30413629 PMC6282239

[39] Murray LS , Lu Y , Taggart A , et al . Chemical chaperone treatment reduces intracellular accumulation of mutant collagen IV and ameliorates the cellular phenotype of a COL4A2 mutation that causes haemorrhagic stroke. Hum Mol Genet 2014;23:283–92. 10.1093/hmg/ddt418 24001601 PMC3869351

