# Supplementary material for: High frequency of HTRA1 AND ABCC6 mutations in Japanese patients with adult-onset cerebral small vessel disease
Source: J Neurol Neurosurg Psychiatry. 2022 Oct 19;94(1):74–81. doi: 10.1136/jnnp-2022-329917 (PMC9763231; doi:10.1136/jnnp-2022-329917)
Supplement: Supplementary data [file jnnp-2022-329917supp001.pdf]

SUPPLEMENTARY METHODS

Measuring the protease activity of novel mutant HTRA1 proteins

The detailed protocols for measuring HTRA1 protease activity have been previously reported.[1] Briefly, an expression plasmid for each *HTRA1* complementary DNA (cDNA) variant was generated. The plasmid vectors were transfected into FreeStyle 293 cells (Thermo Fisher Scientific). After incubation, secreted HTRA1 proteins were purified from the culture medium. After pre-incubating 1 μg of the recombinant HTRA1 protein, the protease activities of these HTRA1 proteins were evaluated using fluorescein isothiocyanate (FITC)-labeled casein as a substrate (Fluorescent Protease Assay Kit; Pierce, Rockford, IL, USA). Wild-type (WT) and S328A were used as positive and negative controls, respectively. S328A is a mutant HTRA1 protein that inhibits protease activity. The protease activities of mutant HTRA1 proteins and the positive and negative controls were calculated from the slope of the linear portion of normalized fluorescence vs. time plots at 30 min, 60 min, and 90 min. The amount of HTRA1 protein was analyzed by sodium dodecyl sulfate-polyacrylamide

gel electrophoresis (SDS-PAGE) and stained with SYPROR©Ruby Protein Gel Stain (Thermo Fisher Scientific, MA, USA).

WES protocols

Exome analysis was conducted using an outsourcing service (Macrogen, Korea).

The SureSelect Human All Exon v6 Kit (Agilent Technologies, Santa Clara, CA, USA) was used as a capture kit. The next-generation sequencer was run on an Illumina HiSeq 4000 or NovaSeq6000 (Illumina, San Diego, CA) using 150 bp paired-end reads. The raw sequence was mapped to the human genome using the Burrows-Wheeler alignment (BWA) tool. A SAM file was generated using Picard. Picard was used to detect but not remove duplicate reads. Variant calling and annotation were performed using GATK (http://www.broadinstitute.org). org/gatk/) and SnpEff (http://snpeff.sourceforge.net/download.html). The hg19 data from the UCSC and GRCh38 data from the NCBI were used as the reference genome data for 53 and 10 samples, respectively.

Detecting the CNV of causative CSVD genes

We investigated the binary alignment map (BAM) files of WES through cn.MOPS using R software (ver. 4.1.0) to identify *HTRA1*, *COL4A1/A2,* and *ABCC6* CNVs among patients without mutations in causative CSVD genes or patients with heterozygous mutations in *ABCC6* or *COL4A2.* cn.MOPS is an algorithm for detecting CNVs from BAM files of next-generation sequencing.[2] The R package cn.MOPS is available on the website (http://www.bioinf.jku.at/software/cnmops/). The minWidth and minReadCount parameters were set to 1 and 100, respectively, and the other cn.MOPS parameters did not change. The read count ratio and estimation of the copy number of each *HTRA1*, *COL4A1/A2,* and *ABCC6* exon were calculated according to the cn.MOPS algorithm.

A ddPCR was performed to verify the cn.MOPS-identified CNVs. We used forward and reverse primers and fluorescent probes for exon 25 of *ABCC6* and *BCKDHA* on genomic DNA as a reference.[3] Probes for exon 25 of *ABCC6* and *BCKDHA* in genomic DNA were labeled with FAM and HEX, respectively. The

forward primer, reverse primer, and probe sequences were 5’-GCAAAGCCCACCTCAGT-3’, 5’-AGTCTCTGCCTCTGTCTGT-3’, and 5’-56-FAM/CTCGTGGGC/Zen/TTCTCTGTCTCTGCT/3IABkFQ, respectively. After the primer and probe of exon 25 of *ABCC6* and *BCKDHA* were mixed with ddPCR Supermix for Probes (No dUTP) (Bio-Rad, CA, USA), droplets were generated using a Bio-Rad QX200 Droplet Generator (Bio-Rad, CA, USA). PCR was then performed. The ddPCR cycling parameters were as follows: initial denaturation for 10 min at 95°C, 40 cycles of 30 s at 94°C, 2 min at 55°C, and final elongation for 10 min at 98°C. We calculated the fluorescence value of FAM/HEX and the copy number of *ABCC6* exon 25 using a Bio-Rad QX200 Droplet Reader (Bio-Rad, CA, USA).

SUPPLEMENTARY STATISTICAL ANALYSIS

Protease activity of each HTRA1 protein

The protease activity of the HTRA1 proteins was statistically analyzed using MATLAB R2020b Update 3 (9.9.0.1538559). Groups were compared using an

ANOVA for independent samples, followed by Bonferroni's correction when the overall p-value was < 0.05.

Logistic regression analysis to investigate the association of Group1 mgCSVD with clinical and imaging features using stepwise selection methods

We performed logistic regression analysis using forward-backward stepwise selection methods to verify independent factors significantly associated with mgCSVD in Group1. Exploratory variables included family history (first relative, second relative, or first and/or second relatives), sex, vascular risk factors (HT, diabetes mellitus (DM), dyslipidemia (DL)), clinical symptoms or signs (stroke, CI/dementia, and GD), neuroimaging findings (positive LI, multiple LIs, severe ECLs, and severe ATLs), and age of onset of neurological findings/signs. Optimal exploratory variables were selected by calculating the Akaike information criterion. Statistical analyses were performed using R (version 4.1.2) in RStudio (2021.09.1).

SUPPLEMENTARY RESULTS

Protease activity of novel mutant HTRA1 proteins

Protease activities of L253R and V279M proteins were significantly decreased compared to those of the WT (Supplementary Figure 2).

Detecting *ABCC6* CNVs

The cn.MOPS results indicated that one patient without mutations in any causative CSVD gene had one copy of exon 7–10, 16–17, and 21–25 of *ABCC6*. None of the patients with heterozygous mutations in *ABCC6* or *COL4A2* had *HTRA1*, *COL4A1/A2,* and *ABCC6* CNVs.

The results of ddPCR also indicated one copy of exon 25 of *ABCC6* in this patient (Supplementary Figure 3).

Logistic regression analysis to investigate the association of Group1 mgCSVD with clinical and imaging features using stepwise methods

After the logistic regression analysis using stepwise methods, the family history of first relatives (p = 0.004), HT (p = 0.005), and multiple LIs (p = 0.003) remained significant (Supplementary Table 3).

Supplementary Table 1. Hospitals participated in this study

Hospital	Number of patients
Department of Behavioral Neurology & Cognitive Neuroscience, Tohoku University Graduate School of Medicine	2
Department of Cerebrovascular Medicine, Kohnan Hospital	1
Department of Cerebrovascular Medicine, Steel Memorial Yawata Hospital	1
Department of Neurology, Anjo Kosei Hospital	1
Department of Neurology, Asoka Hospital	1
Department of Neurology, Brain Research Institutes, Niigata University	11
Department of Neurology, Central Hospital Hyogo Rehabilitation Center	2
Department of Neurology, Chiba University Hospital	2
Department of Neurology, Chikamori Hospital	1
Department of Neurology, Ebara Hospital	1
Department of Neurology, Ehime Prefectural Central Hospital	1
Department of Neurology, Fukuoka Mirai Hospital	1
Department of Neurology, Fukuoka University Hospital	1
Department of Neurology, General Tokyo Hospital	1
Department of Neurology, Gunma University Hospital	1
Department of Neurology, Hiroshima Red Cross Hospital & Atomic-bomb Survivors Hospital	1
Department of Neurology, Hitachinaka General Hospital	1
Department of Neurology, Japanese Red Cross Nagoya Daini Hospital	1
Department of Neurology, Japanese Red Cross Osaka Hospital	6
Department of Neurology, Kagawa Prefectural Central Hospital	1
Department of Neurology, Kanazawa Medical University	1
Department of Neurology, Kanazawa University Hospital	3
Department of Neurology, Kawasaki Medical School	2
Department of Neurology, Keio University Hospital	2
Department of Neurology, Kido Hospital	1
Department of Neurology, Kitamurayama Hospital	1

Department of Neurology, Kobe city Medical Center General Hospital	1
Department of Neurology, Kyoto Prefectural University of Medicine	1
Department of Neurology, Mie University Hospital	1
Department of Neurology, Minami-Okayama Medical Center	1
Department of Neurology, Murakami General Hospital	2
Department of Neurology, Nagano Red Cross Hospital	1
Department of Neurology, Nagaoka Red Cross Hospital	4
Department of Neurology, Nagoya University Hospital	1
Department of Neurology, Nakamura Memorial Hospital	1
Department of Neurology, Nara Medical University Hospital	1
Department of Neurology, National Cerebral and Cardiovascular Center	1
Department of Neurology, National Hakone Hospital	1
Department of Neurology, Niigata City General Hospital	7
Department of Neurology, Niigata Prefectural Shibata Hospital	1
Department of Neurology, Nippon Medical School Musashi Kosugi Hospital	1
Department of Neurology, Okayama Kyokuto Hospotal	2
Department of Neurology, Okayama University Hospital	2
Department of Neurology, Osaka City General Hospital	1
Department of Neurology, Osaka City University Hospital	2
Department of Neurology, Osaka General Medical Center	1
Department of Neurology, Osaka University Hospital	3
Department of Neurology, Osaka city Kosai-in fuzoku hospital	1
Department of Neurology, Public Central Hospital of Matto Ishikawa	1
Department of Neurology, Saiki Hospital	1
Department of Neurology, Saitama Medical University International Medical Center	1
Department of Neurology, Shiseikai Daini Hospital	2
Department of Neurology, Showa General Hospital	1
Department of Neurology, Soseikai General Hospital	1
Department of Neurology, Southern Tohoku General Hospital	1
Department of Neurology, Takasaki General Medical Center	1
Department of Neurology, Tane Second Hospital	1

Department of Neurology, The Jikei University Hospital	1
Department of Neurology, Tokyo Metropolitan Bokutoh Hospital	1
Department of Neurology, Tokyo National Hospital	1
Department of Neurology, Tokyo Women's Medical University Hospital	1
Department of Neurology, Toranomon Hospital	2
Department of Neurology, Toyama Rosai Hospital	1
Department of Neurology, Tsubame Rosai Hospital	1
Department of Neurology, University of Tsukuba Hospital	1
Department of Neurosciences, Tane General Hospital	1
Division of Gastroenterology, Hepato-biliary-pancreatology and Neurology, Akita University School of Medicine	1
Kitakanto Neurologic Disorders Research Center, Shinozuka Hospital	2
Stroke Center, Kyorin University Hospital	1

Supplementary Table 1. Hospitals participated in this study.

BMJ Publishing Group Limited (BMJ) disclaims all liability and responsibility arising from any reliance placed on this supplemental material which has been supplied by the author(s)

Supplementary Table 2. Basic information of CSVD patients with age of onset of stroke, GD and/or CI/dementia ≤ and > 55 years old.

Items	Age of onset ≤ 55 years old (Group1)	Age of onset > 55 years old (Group2)
	n = 75	n = 31
Family history		
First relatives (%), NA	34 (45.3), 0	25 (80.6), 0
Second relatives (%), NA	17 (22.7), 0	16 (51.6), 0
First and/or second relatives (%), NA	41 (54.7), 0	31 (100), 0
Consanguity marriage of parents (%), NA	6 (8), 0	4 (12.9), 0
Male (%), NA	53 (70.7), 0	16 (51.6), 0
Risk factors		
HT (%), NA	36 (48), 0	21 (67.7), 0
DM (%), NA	6 (8), 0	5 (16.1), 0
DL (%), NA	28 (37.3), 0	10 (32.3), 0
Alcohol (%), NA	6 (8.2), 2	6 (20), 1
Smoking (%), NA	31 (43.7), 4	10 (33.3), 1
Neurological symptoms/signs		
CI (MoCA-J ≤ 26) (%), NA	64 (92.8), 6	22 (81.5), 4
Dementia (%), NA	49 (65.3), 0	19 (61.3), 0
CI and/or dementia (%), NA	69 (92), 0	27 (87.1), 0
GD (%), NA	47 (62.7), 0	22 (71), 0
Stroke (%), NA	45 (60), 0	14 (45.2), 0
Migraine (%), NA	17 (23.3), 2	7 (22.6), 0
Pseudobulbar palsy (%), NA	26 (35.6), 2	9 (29), 0

Extraneurological symptoms/signs		
Alopecia (%), NA	18 (26.1), 6	3 (10.3), 2
Lumbago and/or spondylosis deformans (%), NA	33 (45.2), 2	13 (41.9), 0
Hemorrhage in fundus (%), NA	4 (8.7), 29	1 (6.3), 15
Brain imaging findings		
Fazekas grade of WMHs		
Grade 3 (PVH) (%), NA	69 (92), 0	31 (100), 0
Grade III (DWMH) (%), NA	75 (100), 0	30 (96.8), 0
LI		
Positive LI (%), NA	63 (84), 0	25 (80.6), 0
at semiovale (%), NA	52 (69.3), 0	18 (58.1), 0
at BG (%), NA	33 (44), 0	14 (45.2), 0
at thalamus (%), NA	41 (54.7), 0	13 (41.9), 0
at brainstem (%), NA	26 (34.7), 0	6 (19.4), 0
at cerebellum (%), NA	8 (10.7), 0	2 (6.5), 0
Multiple LIs (%), NA	59 (78.7), 0	21 (67.7), 0
dPVS (%), NA	72 (97.3), 1	31 (100), 0
Severe ECL (%), NA	39 (52), 0	18 (58.1), 0
Severe ATL (%), NA	21 (28), 0	4 (12.9), 0
MBs		
Positive MBs (%), NA	48 (73.8), 10	20 (74.1), 4
Multiple MBs (%), NA	42 (64.6), 10	13 (48.1), 4
Strict lobar (%), NA	2 (3.1), 10	2 (7.4), 4
Non lobar (%), NA	9 (13.8), 10	4 (14.8), 4
Age of genetic test (years old)		
mean ± sd	52.2 ± 8.6	65.8 ± 6.0
median (minimum-maximum)	53 (32-79)	64 (56-79)
Age of onset of stroke, GD and/or CI/dementia (years old)		
mean ± sd	46.0 ± 7.5	61.4 ± 4.0

BMJ Publishing Group Limited (BMJ) disclaims all liability and responsibility arising from any reliance placed on this supplemental material which has been supplied by the author(s)

median (minimum-maximum)	48 (23-55)	60 (56-70)

Supplementary Table 2. Basic information of CSVD patients with age of onset of stroke, GD and/or CI/dementia ≤ and > 55 years old.

CSVD indicates cerebral small vessel disease; HT, hypertension; DM, diabetes mellitus; DL, dyslipidemia; MoCA-J, Montreal cognitive assessment battery in Japanese edition; CI, cognitive impairment; GD, gait disturbance; WMH, white matter hyperintensity; PVH, periventricular hyperintensity; DSWMH, deep and subcortical white matter hyperintensity; LI, lacunar infarction; dPVS, dilated perivascular spaces; ECL, external capsular lesion; ATL, anterior temporal lesion; MBs, microbleeds; sd, standard deviations; NA, number of patients who was not available.

Supplementary Table 3. Summary of identified mutations.

cDNA, p.AA	SNP ID	Exon	n, Group1	n, Group2	MAF of gnomAD	In Silico				ACMG				
						PolyPhen2	SIFT	Provean	CADD PHRED	PVS1	PS1-4	PM1-6	PP1-5	Classification
NOTCH3														
c.163T>C, p.C55R	NA	2	1	0	NA	probably damaging	DELETERIOUS	Deleterious	27	-	-	1, 2	1, 2, 3, 4	Likely Pathogenic
c.224G>C, p.R75P	NA	3	1	1	NA	probably damaging	TOLERATED	Deleterious	20.9	-	-	1, 2	1, 2, 3, 4	Likely Pathogenic
c.397C>T, p.R133C	rs137852642	4	1	0	0.000004515	probably damaging	TOLERATED	Deleterious	28.8	-	-	1, 2	1, 2, 3, 4	Likely Pathogenic
c.544C>T, p.R182C	rs28933697	4	1	0	0.00003185	probably damaging	DELETERIOUS	Deleterious	29.5	-	-	1, 2	1, 2, 3, 4	Likely Pathogenic
c.548G>C, p.C183S	NA	4	1	1	NA	probably damaging	DELETERIOUS	Deleterious	26.2	-	-	1, 2	1, 2, 3, 4	Likely Pathogenic
c.635G>A, p.C212Y	NA	4	1	0	NA	probably damaging	DELETERIOUS	Deleterious	24.4	-	-	1, 2	1, 2, 3, 4	Likely Pathogenic
c.953G>A, p.C318Y	NA	6	1	1	NA	probably damaging	DELETERIOUS	Deleterious	26.8	-	-	1, 2	1, 2, 3, 4	Likely Pathogenic

c.994C>T, p.R332C	NA	6	1	1	NA	probably damaging	TOLERATED	Deleterious	31	-	-	1, 2	1, 2, 3, 4	Likely Pathogenic
c.1063T>G, p.C355G	NA	7	1	0	NA	probably damaging	DELETERIOUS	Deleterious	28.5	-	-	1, 2	2, 3, 4	Likely Pathogenic
c.1249T>A, p.C417S	NA	8	1	0	NA	probably damaging	DELETERIOUS	Deleterious	27.5	-	-	1, 2	2, 3, 4	Likely Pathogenic
c.1279C>T, p.R427C	NA	8	1	0	NA	probably damaging	DELETERIOUS	Deleterious	32	-	-	1, 2	1, 2, 3, 4	Likely Pathogenic
c.1337G>T, p.C446F	NA	8	1	0	NA	probably damaging	DELETERIOUS	Deleterious	26	-	-	1, 2	1, 2, 3, 4	Likely Pathogenic
c.1501G>T, p.G501C	NA	10	1	0	NA	probably damaging	DELETERIOUS	Deleterious	26.5	-	-	1, 2	2, 3, 4	Likely Pathogenic
c.1630C>T, p.R544C	rs201118034	11	1	1	0.0002947	possibly damaging	TOLERATED	Deleterious	23.1	-	-	1, 2	1, 2, 4	Likely Pathogenic
c.1672C>T, p.R558C	rs75068032	11	1	0	0.00003187	probably damaging	TOLERATED	Deleterious	29.4	-	-	1, 2	1, 2, 3, 4	Likely Pathogenic
c.1789T>A, p.C597S	NA	11	0	1	NA	probably damaging	DELETERIOUS	Deleterious	27.5	-	-	1, 2	1, 2, 3, 4	Likely Pathogenic
c.1819C>T, p.R607C	rs777751303	11	1	0	0.000003998	probably damaging	TOLERATED	Deleterious	25.1	-	-	1, 2	1, 2, 3, 4	Likely Pathogenic

c.2149C>T, p.R717C	rs144163298	14	0	1	0.00003637	probably damaging	DELETERIOUS	Deleterious	25.7	-	-	1, 2	1, 2, 3, 4	Likely Pathogenic
c.2861A>G, p.Y954C	NA	18	1	0	NA	probably damaging	DELETERIOUS	Deleterious	29	-	-	1, 2	2, 3, 4	Likely Pathogenic
c.3010T>G, p.C1004G	NA	19	1	0	NA	probably damaging	TOLERATED	Deleterious	28.5	-	-	1, 2	1, 2, 3, 4	Likely Pathogenic
c.3062A>G, p.Y1021C	rs1167405466	19	2	0	0.00003187	possibly damaging	TOLERATED	Deleterious	23.2	-	-	1, 2	1, 2, 4	Likely Pathogenic
c.3091C>T, p.R1031C	NA	19	1	0	NA	possibly damaging	TOLERATED	Deleterious	23.4	-	-	1, 2	1, 2, 4	Likely Pathogenic
c.3226C>T, p.R1076C	rs1438626607	20	1	0	0.00003194	probably damaging	DELETERIOUS	Deleterious	26.5	-	-	1, 2	1, 2, 3, 4	Likely Pathogenic
c.3427C>T, p.R1143C	rs60373464	21	1	0	0.00001769	probably damaging	TOLERATED	Deleterious	23.7	-	-	1, 2	1, 2, 3, 4	Likely Pathogenic
c.4111C>T, p.R1371C	NA	24	1	0	NA	probably damaging	TOLERATED	Deleterious	24.5	-	-	1, 2	2, 3, 4	Likely Pathogenic
HTRA1														
c.496C>T, p.R166C	NA	2	1	0	NA	probably damaging	DELETERIOUS	Deleterious	28.8	-	1, 3	1, 2	1, 3, 4	Pathogenic

c.497G>T, p.R166L	NA	2	1	0	NA	probably damaging	DELETERIOUS	Deleterious	32	-	1, 3	1, 2	1, 3, 4	Pathogenic
c.758T>G, p.L253R	NA	3	1	0	NA	probably damaging	DELETERIOUS	Deleterious	24.9	-	3	1, 2	2	Likely Pathogenic
c.835G>A, p.V279M	rs745305935	4	1	0	0.000003984	probably damaging	DELETERIOUS	Deleterious	28.5	-	3	1	3, 4	Likely Pathogenic
c.854C>T, p.P285L	rs587776446	4	1	0	0.00000398	probably damaging	DELETERIOUS	Deleterious	29.4	-	3	1	1, 3, 4	Likely Pathogenic
c.889G>A, p.V297M	NA	4	1	0	NA	probably damaging	DELETERIOUS	Deleterious	31	-	3	1, 2	1, 3, 4	Pathogenic
c.904C>T, p.R302X	rs113993970	4	2	1	0.00001062	NA	NA	NA	40	1	1	1	1	Pathogenic
c.905G>A, p.R302Q	NA	4	2	0	NA	probably damaging	DELETERIOUS	Deleterious	32	-	3	1, 2	1, 3, 4	Pathogenic
ABCC6														
c.41G>A, p.W14X	NA	2	1	0	NA	NA	NA	NA	36	1	-	2, 4	-	Pathogenic
c.1132C>T, p.Q378X	rs72650699	9	1	0	0.00006017	NA	NA	NA	37	1	-	4	1	Pathogenic
c.2542delA, p.V848fs	rs67867306	19	4	0	0.0002015	NA	NA	NA	NA	1	-	4	1	Pathogenic
c.3936_3937insG, p.L1313fs	NA	28	1	0	NA	NA	NA	NA	NA	1	-	2, 4	1	Pathogenic

Deletion of exons	NA	7-10, 16-17, 21-25	0	1	NA	NA	NA	NA	NA	1		2, 4	-	Pathogenic
COL4A1														
c.*33T>A	NA		1	0	NA	NA	NA	NA	19.89	-	3	2	-	Undetermined significance
COL4A2														
c.3527_3529delGAG, p.Gly1176del	NA	19	1	0	NA	NA	NA	NA	NA	-	-	1, 4	-	Undetermined significance
TREX1														
c.858dupG, p.L287LAfs*38	NA	2	1	0	NA	NA	NA	NA	NA	1	-	1, 2, 4	-	Likely Pathogenic

Supplementary Table 3. Summary of identified mutations.

AA indicates amino acids; MAF, minor allele frequency; gnomAD, genome aggregation database; CADD, combined annotation dependent depletion; ACMG, American College of Medical Genetics and Genomics; NA, not available.

Supplementary Table 4. Summary of non-CADASIL mgCSVD

Non-CADASIL mgCSVD	Heterozygous *HTRA1*	CARASIL	PXE	Heterozygous *ABCC6*	*COL4A1*	*COL4A2*	RVCL
	n = 9	n = 2	n = 3	n = 3	n = 1	n = 1	n = 1
Family history							
First relatives (%), NA	7 (77.8), 0	0 (0), 0	2 (66.7), 0	1 (33.3), 0	0 (0), 0	1 (100), 0	1 (100), 0
Second relatives (%), NA	1 (11.1), 0	0 (0), 0	2 (66.7), 0	0 (0), 0	0 (0), 0	0 (0), 0	1 (100), 0
First and/or second relatives (%), NA	7 (77.8), 0	0 (0), 0	2 (66.7), 0	1 (33.3), 0	0 (0), 0	1 (100), 0	1 (100), 0
Consanguity marriage of parents (%), NA	0 (0), 0	0 (0), 0	0 (0), 0	1 (33.3), 0	0 (0), 0	0 (0), 0	0 (0), 0
Male (%), NA	6 (66.7), 0	1 (50), 0	2 (66.7), 0	2 (66.7), 0	1 (100), 0	0 (0), 0	1 (100), 0
Risk factors							
HT (%), NA	4 (44.4), 0	0 (0), 0	2 (66.7), 0	1 (33.3), 0	0 (0), 0	1 (100), 0	0 (0), 0
DM (%), NA	0 (0), 0	0 (0), 0	1 (33.3), 0	1 (33.3), 0	0 (0), 0	1 (100), 0	0 (0), 0
DL (%), NA	4 (44.4), 0	0 (0), 0	0 (0), 0	0 (0), 0	0 (0), 0	0 (0), 0	0 (0), 0
Alcohol (%), NA	0 (0), 0	0 (0), 0	0 (0), 0	0 (0), 0	0 (0), 1	0 (0), 0	0 (0), 0
Smoking (%), NA	2 (22.2), 0	1 (50), 0	2 (66.7), 0	1 (33.3), 0	0 (0), 1	0 (0), 0	0 (0), 0

Neurological symptoms/signs							
CI (MoCA-J ≤ 26) (%), NA	7 (77.8), 0	2 (100), 0	3 (100), 0	2 (100), 1	0 (0), 1	1 (100), 0	1 (100), 0
Dementia (%), NA	6 (66.7), 0	2 (100), 0	2 (66.7), 0	3 (100), 0	1 (100), 0	0 (0), 0	1 (100), 0
CI and/or dementia (%), NA	7 (77.8), 0	2 (100), 0	3 (100), 0	3 (100), 0	1 (100), 0	1 (100), 0	1 (100), 0
GD (%), NA	7 (77.8), 0	2 (100), 0	1 (33.3), 0	2 (66.7), 0	1 (100), 0	1 (100), 0	1 (100), 0
Stroke (%), NA	7 (77.8), 0	0 (0), 0	2 (66.7), 0	1 (33.3), 0	1 (100), 0	0 (0), 0	1 (100), 0
Migraine (%), NA	3 (37.5), 1	0 (0), 0	2 (66.7), 0	2 (66.7), 0	0 (0), 0	0 (0), 0	0 (0), 0
Pseudobulbar palsy (%), NA	4 (44.4), 0	1 (50), 0	2 (66.7), 0	1 (50), 1	1 (100), 0	0 (0), 0	0 (0), 0
Extraneurological symptoms/signs							
Alopecia (%), NA	3 (37.5), 1	1 (50), 0	2 (100), 1	1 (33.3), 0	0 (0), 0	0 (0), 0	1 (100), 0
Lumbago and/or spondylosis deformans (%), NA	7 (77.8), 0	2 (100), 0	3 (100), 0	1 (33.3), 0	0 (0), 0	0 (0), 0	0 (0), 0
Hemorrhage in fundus (%), NA	0 (0), 6	0 (0), 0	1 (50), 1	0 (0), 2	0 (0), 1	0 (0), 1	0 (0), 0
Brain imaging findings							
Fazekas grade of WMHs							
Grade 3 (PVH) (%), NA	8 (88.9), 0	2 (100), 0	2 (66.7), 0	3 (100), 0	1 (100), 0	1 (100), 0	1 (100), 0
Grade III (DWMH) (%), NA	9 (100), 0	2 (100), 0	3 (100), 0	3 (100), 0	1 (100), 0	1 (100), 0	1 (100), 0
LI							
Positive LI (%), NA	9 (100), 0	2 (100), 0	2 (66.7), 0	2 (66.7), 0	1 (100), 0	0 (0), 0	1 (100), 0
at semiovale (%), NA	8 (88.9), 0	2 (100), 0	1 (33.3), 0	1 (33.3), 0	0 (0), 0	0 (0), 0	1 (100), 0

at BG (%), NA	3 (33.3), 0	0 (0), 0	1 (33.3), 0	1 (33.3), 0	1 (100), 0	0 (0), 0	0 (0), 0
at thalamus (%), NA	3 (33.3), 0	1 (50), 0	1 (33.3), 0	1 (33.3), 0	1 (100), 0	0 (0), 0	0 (0), 0
at brainstem (%), NA	1 (11.1), 0	2 (100), 0	2 (66.7), 0	1 (33.3), 0	0 (0), 0	0 (0), 0	0 (0), 0
at cerebellum (%), NA	0 (0), 0	0 (0), 0	1 (33.3), 0	0 (0), 0	0 (0), 0	0 (0), 0	0 (0), 0
Multiple LIs (%), NA	8 (88.9), 0	2 (100), 0	2 (66.7), 0	2 (66.7), 0	1 (100), 0	0 (0), 0	1 (100), 0
dPVS (%), NA	9 (100), 0	2 (100), 0	3 (100), 0	3 (100), 0	1 (100), 0	0 (0), 0	1 (100), 0
Severe ECL (%), NA	7 (77.8), 0	2 (100), 0	1 (33.3), 0	0 (0), 0	1 (100), 0	0 (0), 0	0 (0), 0
Severe ATL (%), NA	0 (0), 0	1 (50), 0	0 (0), 0	0 (0), 0	1 (100), 0	0 (0), 0	0 (0), 0
MBs							
Positive MBs (%), NA	5 (71.4), 2	2 (100), 0	2 (100), 1	1 (50), 1	1 (100), 0	0 (0), 0	1 (100), 0
Multiple MBs (%), NA	4 (57.1), 2	2 (100), 0	2 (100), 1	1 (50), 1	1 (100), 0	0 (0), 0	1 (100), 0
Strict lobar (%), NA	1 (14.3), 2	0 (0), 0	0 (0), 1	1 (50), 1	0 (0), 0	0 (0), 0	0 (0), 0
Non lobar (%), NA	0 (0), 2	0 (0), 0	0 (0), 1	1 (50), 1	0 (0), 0	0 (0), 0	1 (100), 0
Age of genetic test (years old)							
mean ± sd	57.6 ± 11.1	40.0 ± 9.9	47.7 ± 13.5	52.0 ± 12.1	51	63	41
median (minimum-maximum)	58 (32-71)	40 (33-47)	48 (34-61)	45 (45-66)	-	-	-
Age of onset of stroke, GD and/or CI/dementia (years old)							
mean ± sd	50.4 ± 9.4	36.5 ± 9.2	42.7 ± 7.8	45 ± 19.7	30	45	33
median (minimum-maximum)	51 (32-68)	36.5 (30-43)	45 (34-49)	42 (27-66)	-	-	-

Supplementary table 4. Summary of non-CADASIL mgCSVD

mgCSVD indicates monogenic cerebral small vessel disease; HT, hypertension; DM, diabetes mellitus; DL, dyslipidemia; MoCA-J, Montreal cognitive assessment battery in Japanese edition; CI, cognitive impairment; GD, gait disturbance; WMH, white matter hyperintensity; PVH, periventricular hyperintensity; DSWMH, deep and subcortical white matter hyperintensity; LI, lacunar infarction; dPVS, dilated perivascular spaces; ECL, external capsular lesion; ATL, anterior temporal lesion; MBs, microbleeds; sd, standard deviations; NA, number of patients who was not available; Heterozygous *HTRA1*, heterozygous *high-temperature requirement A serine peptidase 1 (HTRA1)*-related CSVD, CARASIL, cerebral autosomal recessive arteriopathy with subcortical infarcts and leukoencephalopathy; PXE, pseudoxanthoma elasticum; Heterozygous *ABCC6*, heterozygous mutation in *ATP Binding Cassette Subfamily C Member 6 (ABCC6)*; *COL4A1*, *COL4A1*-related CSVD; *COL4A2*, *COL4A2*-related CSVD; RVCL, retinal vasculopathy with cerebral leukoencephalopathy.

Supplemental material BMJ Publishing Group Limited (BMJ) disclaims all liability and responsibility arising from any reliance placed on this supplemental material which has been supplied by the author(s) J Neurol Neurosurg Psychiatry

Supplementary Table 5. Comparison between mgCSVD including CADASIL and non-CADASIL mgCSVD and undetermined in Group1

	mgCSVD	CADASIL	Non-CADASIL mgCSVD	Undetermined	p-value, 2 groups	p-value, 3 groups
					mgCSVD vs undetermined	CADASIL vs non-CADASIL vs undetermined
n	n = 41	n = 23	n = 18	n = 34		
Family History						
First relatives (%), NA	25 (61.0), 0	15 (65.2), 0	10 (55.6), 0	9 (26.5), 0	0.0028	0.0095
Second relatives (%), NA	10 (24.4), 0	6 (26.1), 0	4 (22.2), 0	7 (20.6), 0	0.6954	0.8872
First and/or second relatives (%), NA	27 (65.9), 0	17 (73.9), 0	10 (55.6), 0	14 (41.2), 0	0.0326	0.0513
Consanguity marriage of parents (%), NA	3 (7.3), 0	2 (8.7), 0	1 (5.6), 0	3 (8.8), 0	0.8108	0.9082
Male (%), NA	28 (68.3), 0	16 (69.6), 0	12 (66.7), 0	25 (73.5), 0	0.62	0.8664
Risk factors						
HT (%), NA	14 (34.1), 0	7 (30.4), 0	7 (38.9), 0	22 (64.7), 0	0.0084	0.0267
DM (%), NA	4 (9.8), 0	2 (8.7), 0	2 (11.1), 0	2 (5.9), 0	0.5382	0.7949
DL (%), NA	15 (36.6), 0	11 (47.8), 0	4 (22.2), 0	13 (38.2), 0	0.8831	0.2404
Alcohol (%), NA	2 (5.0), 1	2 (8.7), 0	0 (0), 1	4 (12.1), 1	0.2702	0.3336
Smoking (%), NA	16 (41.0), 2	10 (45.5), 1	6 (35.3), 1	15 (46.9), 2	0.621	0.7236
Neurological symptoms/signs						
CI (MoCA-J ≤ 26) (%), NA	35 (89.7), 2	20 (87.0), 0	15 (93.8), 2	29 (96.7), 4	0.2715	0.3951
Dementia (%), NA	28 (68.3), 0	14 (60.9), 0	14 (77.8), 0	21 (61.8), 0	0.5543	0.4439
CI and/or dementia (%), NA	37 (90.2), 0	20 (87), 0	17 (94.4), 0	32 (94.1), 0	0.5382	0.5632
GD (%), NA	23 (56.1), 0	9 (39.1), 0	14 (77.8), 0	24 (70.6), 0	0.1965	0.0173
Stroke (%), NA	25 (61.0), 0	15 (65.2), 0	10 (55.6), 0	20 (58.8), 0	0.8498	0.8071
Migraine (%), NA	9 (22.5), 1	3 (13), 0	6 (35.3), 1	8 (24.2), 1	0.8608	0.2541
Pseudobulbar palsy (%), NA	13 (33.3), 2	4 (18.2), 1	9 (52.9), 1	13 (38.2), 0	0.6626	0.0727
Extraneurological symptoms/signs						
Alopecia (%), NA	9 (23.7), 3	2 (9.1), 1	7 (43.8), 2	9 (29), 3	0.6148	0.0492
Lumbago and/or spondylosis deformans (%), NA	17 (43.6), 2	5 (23.8), 2	12 (66.7), 0	16 (47.1), 0	0.7664	0.0263
Hemorrhage in fundus (%), NA	2 (8.3), 17	1 (6.3), 7	1 (12.5), 10	2 (9.1), 12	0.7898	0.9168

Brain imaging findings						
Fazekas grade of WMHs						
Grade 3 (PVH) (%), NA	37 (90.2), 0	21 (91.3), 0	16 (88.9), 0	32 (94.1), 0	0.5382	0.7949
Grade III (DWMH) (%), NA	41 (100), 0	23 (100), 0	18 (100), 0	34 (100), 0	-	-
LI						
Positive LI (%), NA	38 (92.7), 0	23 (100), 0	15 (83.3), 0	25 (73.5), 0	0.0243	0.0279
at semiovale (%), NA	30 (73.2), 0	19 (82.6), 0	11 (61.1), 0	22 (64.7), 0	0.4287	0.244
at BG (%), NA	17 (41.5), 0	12 (52.2), 0	5 (27.8), 0	16 (47.1), 0	0.627	0.2625
at thalamus (%), NA	21 (51.2), 0	14 (60.9), 0	7 (38.9), 0	20 (58.8), 0	0.5102	0.3009
at brainstem (%), NA	15 (36.6), 0	9 (39.1), 0	6 (33.3), 0	11 (32.4), 0	0.7014	0.8621
at cerebellum (%), NA	5 (12.2), 0	4 (17.4), 0	1 (5.6), 0	3 (8.8), 0	0.6377	0.4261
Multiple LIs (%), NA	36 (87.8), 0	21 (91.3), 0	15 (83.3), 0	23 (67.6), 0	0.0339	0.087
dPVS (%), NA	40 (97.6), 0	23 (100), 0	17 (94.4), 0	32 (97.0), 1	0.8761	0.5462
Severe ECL (%), NA	25 (61), 0	15 (65.2), 0	10 (55.6), 0	14 (41.2), 0	0.0875	0.1924
Severe ATL (%), NA	10 (24.4), 0	9 (39.1), 0	1 (5.6), 0	11 (32.4), 0	0.4445	0.0444
MBs						
Positive MBs (%), NA	29 (80.6), 5	17 (81.0), 2	12 (80.0), 3	19 (65.5), 5	0.1178	0.2936
Multiple MBs (%), NA	24 (66.7), 5	13 (61.9), 2	11 (73.3), 3	18 (62.1), 5	0.5751	0.6676
Strict lobar (%), NA	2 (5.6), 5	0 (0), 2	2 (13.3), 3	0 (0), 5	0.1899	0.0300
Non lobar (%), NA	8 (22.2), 5	6 (28.6), 2	2 (13.3), 3	1 (3.4), 5	0.026	0.0354
Age of genetic test (years old)						
mean ± sd	53.8 ± 10.1	56.1 ± 9.0	50.8 ± 10.9	50.4 ± 6.0	0.0457	0.0335
median (minimum-maximum)	54 (32-79)	57 (40-79)	52.5 (32-69)	51 (38-64)		
Age of onset of stroke, GD and/or CI/dementia (years old)						
mean ± sd	45.4 ± 8.0	47.7 ± 6.2	42.4 ± 9.1	46.8 ± 6.8	1	0.0551
median (minimum-maximum)	48 (27-55)	50 (31-55)	44.5 (27-54)	48 (23-55)		

Supplementary Table 5. Comparison between mgCSVD including CADASIL and non-CADASIL mgCSVD and undetermined in Group1

Monogenic indicates the group of patients with monogenic cerebral small vessel disease; mgCSVD, monogenic cerebral small vessel disease; CADASIL, cerebral

autosomal dominant arteriopathy with subcortical infarct and leukoencephalopathy; Undetermined, the group of patients with undetermined cause; HT, hypertension; DM, diabetes mellitus; DL, dyslipidemia; MoCA-J, Montreal cognitive assessment battery in Japanese edition; CI, cognitive impairment; GD, gait disturbance; WMH, white matter hyperintensity; PVH, periventricular hyperintensity; DSWMH, deep and subcortical white matter hyperintensity; LI, lacunar infarction; dPVS, dilated perivascular spaces; ECL, external capsular lesion; ATL, anterior temporal lesion; MBs, microbleeds; sd, standard deviations; NA, number of patients who was not available.

BMJ Publishing Group Limited (BMJ) disclaims all liability and responsibility arising from any reliance placed on this supplemental material which has been supplied by the author(s)

Supplementary Table 6. Logistic regression analysis to investigate association of mgCSVD in Group1 and clinical/imaging findings by using stepwise methods

Items	Odds ratio	95% Confidence interval	p-value
Family history			
first relatives	1.3325	1.0914-1.6268	0.0055
Risk factors			
HT	0.7408	0.6031-0.9098	0.0048
DM	1.5370	1.0496-2.2508	0.0278
Neurological symptoms/signs			
Gait disturbance	0.7470	0.5953-0.9372	0.0125
Neuroimaging findings			
Multiple LIs	1.4184	1.1069-1.8177	0.0064
severe ECLs	1.1866	0.9574-1.4708	0.1163

Supplementary Table 6. Logistic regression analysis to investigate association of mgCSVD in Group1 and clinical/imaging findings by using stepwise methods

mgCSVD indicates monogenic cerebral small vessel disease; HT, hypertension; DM, diabetes mellitus; LI, lacunar infarction; ECL, external capsular lesion.

BMJ Publishing Group Limited (BMJ) disclaims all liability and responsibility arising from any reliance placed on this supplemental material which has been supplied by the author(s)

Supplementary Table 7. Classification of CSVD patients in Group1 by using results of decision tree

Items of classification				
First relatives	Negative			Positive
HT	Positive	Negative		-
Age of onset of stroke, GD and/or CI/dementia	-	> 43 years old	≤ 43 years old	-
Diagnosis				
CADASIL (%)	3 (15.0)	3 (33.3)	2 (16.7)	15 (44.1)
Heterozygous *HTRA1* (%)	1 (5.0)	0	1 (8.3)	6 (17.6)
CARASIL (%)	0	0	2 (16.7)	0
PXE (%)	0	0	1 (8.3)	2 (5.9)
Heterozygous *ABCC6* (%)	0	0	2 (16.7)	0
COL4A1 (%)	0	0	1 (8.3)	0
COL4A2 (%)	0	0	0	1 (2.9)
RVCL (%)	0	0	0	1 (2.9)
Undetermined (%)	16 (80.0)	6 (66.7)	3 (25.0)	9 (26.5)

Supplementary Table 7. Classification of CSVD patients in Group1 by using results of decision tree

CSVD indicated cerebral small vessel disease; HT, hypertension; GD, gait disturbance; CI, cognitive impairment; CADASIL, cerebral autosomal dominant arteriopathy with subcortical infarcts and leukoencephalopathy; Heterozygous *HTRA1*, heterozygous *high-temperature requirement A serine peptidase 1 (HTRA1)*-related CSVD, CARASIL, cerebral autosomal recessive arteriopathy with subcortical infarcts and

BMJ Publishing Group Limited (BMJ) disclaims all liability and responsibility arising from any reliance placed on this supplemental material which has been supplied by the author(s)

leukoencephalopathy; PXE, pseudoxanthoma elasticum; Heterozygous *ABCC6*, heterozygous mutation in *ATP Binding Cassette Subfamily C Member 6 (ABCC6)*; *COL4A1*, *COL4A1*-related CSVD; *COL4A2*, *COL4A2*-related CSVD; RVCL, retinal vasculopathy with cerebral leukoencephalopathy.

Supplementary Table 8. Comparison between mgCSVD and undetermined among patients with age of onset > 55 years old (Group2)

	Monogenic	Undetermined	p-value
n	n=9	n=22	mgCSVD vs Undetermined
Family History			
First relatives (%), NA	8 (88.9), 0	17 (77.3), 0	0.4574
Second relatives (%), NA	5 (55.6), 0	11 (50), 0	0.7787
First and/or second relatives (%), NA	9 (100), 0	22 (100), 0	0
Consanguity marriage of parents (%), NA	0 (0), 0	4 (18.2), 0	0.1705
Male (%), NA	6 (66.7), 0	10 (45.5), 0	0.2834
Risk factors			
HT (%), NA	4 (44.4), 0	17 (77.3), 0	0.0759
DM (%), NA	1 (11.1), 0	4 (18.2), 0	0.6271
DL (%), NA	5 (55.6), 0	5 (22.7), 0	0.0759
Alcohol (%), NA	1 (12.5), 1	5 (22.7), 0	0.5357
Smoking (%), NA	2 (25.0), 1	8 (36.4), 0	0.5593
Neurological symptoms/signs			
CI (MoCA-J ≤ 26) (%), NA	6 (75.0), 1	16 (84.2), 3	0.5737
Dementia (%), NA	5 (55.6), 0	14 (63.6), 0	0.675
CI and/or dementia (%), NA	7 (77.8), 0	20 (90.9), 0	0.3222
GD (%), NA	5 (55.6), 0	17 (77.3), 0	0.2266
Stroke (%), NA	6 (66.7), 0	8 (36.4), 0	0.1238
Migraine (%), NA	2 (22.2), 0	5 (22.7), 0	0.9756
Pseudobulbar palsy (%), NA	3 (33.3), 0	6 (27.3), 0	0.7358
Extraneurological symptoms/signs			
Alopecia (%), NA	2 (22.2), 0	1 (5.0), 2	0.1589

Lumbago and/or spondylosis deformans (%), NA	4 (44.4), 0	9 (40.9), 0	0.8563
Hemorrhage in fundus (%), NA	1 (33.3), 6	0 (0), 9	0.0929
Brain imaging findings			
Fazekas grade of WMHs			
Grade 3 (PVH) (%), NA	9 (100), 0	22 (100), 0	-
Grade III (DWMH) (%), NA	9 (100), 0	21 (95.5), 0	0.5156
LI			
Positive LI (%), NA	8 (88.9), 0	17 (77.3), 0	0.4574
at semiovale (%), NA	8 (88.9), 0	10 (45.5), 0	0.0261
at BG (%), NA	4 (44.4), 0	10 (45.5), 0	0.9591
at thalamus (%), NA	3 (33.3), 0	10 (45.5), 0	0.5347
at brainstem (%), NA	2 (22.2), 0	4 (18.2), 0	0.7961
at cerebellum (%), NA	2 (22.2), 0	0 (0), 0	0.0223
Multiple LIs (%), NA	7 (77.8), 0	14 (63.6), 0	0.4445
dPVS (%), NA	9 (100), 0	22 (100), 0	-
Severe ECL (%), NA	6 (66.7), 0	12 (54.5), 0	0.5347
Severe ATL (%), NA	1 (11.1), 0	3 (13.6), 0	0.849
MBs			
Positive MBs (%), NA	6 (75.0), 1	14 (73.7), 3	0.9432
Multiple MBs (%), NA	5 (62.5), 1	8 (42.1), 3	0.3328
Strict lobar (%), NA	0 (0), 1	2 (10.5), 3	0.3403
Non lobar (%), NA	1 (12.5), 1	3 (15.8), 3	0.8261
Age of genetic test (years old)			
mean ± sd	66.1 ± 5.4	65.7 ± 6.3	0.7102
median (minimum-maximum)	66 (57-73)	64 (56-79)	
Age of onset of stroke, GD and/or CI/dementia (years old)			
mean ± sd	61.1 ± 4.6	61.5 ± 3.9	1
median (minimum-maximum)	58 (57-68)	60.5 (56-70)	

Supplementary Table 8. Comparison between mgCSVD and undetermined among patients with age of onset > 55 years old (Group2)

CADASIL indicates cerebral autosomal dominant arteriopathy with subcortical infarct and leukoencephalopathy; HT, hypertension; DM, diabetes mellitus; DL, dyslipidemia; MoCA-J, Montreal cognitive assessment battery in Japanese edition; CI, cognitive impairment; GD, gait disturbance; WMH, white matter hyperintensity; PVH, periventricular hyperintensity; DSWMH, deep and subcortical white matter hyperintensity; LI, lacunar infarction; dPVS, dilated perivascular spaces; ECL, external capsular lesion; ATL, anterior temporal lesion; MBs, microbleeds; sd, standard deviations; NA, number of patients who was not available.

REFERENCES

1. Uemura M, Nozaki H, Koyama A, et al. *HTRA1* mutations identified in symptomatic carriers have the property of interfering the trimer-dependent activation cascade. *Front Neurol* 2019;10:693.
2. Klambauer G, Schwarzbauer K, Mayr A, et al. cn.MOPS: mixture of Poissons for discovering copy number variations in next-generation sequencing data with a low false discovery rate. *Nucleic Acids Res* 2012;40(9):e69.
3. Zhong Q, Bhattacharya S, Kotsopoulos S, et al. Multiplex digital PCR: breaking the one target per color barrier of quantitative PCR. *Lab Chip* 2011;11(13):2167-74.
4. Iwanaga A, Okubo Y, Yozaki M, et al. Analysis of clinical symptoms and *ABCC6* mutations in 76 Japanese patients with pseudoxanthoma elasticum. *J Dermatol* 2017;44(6):644-50.

SUPPLEMENTARY FIGURES

Supplementary figure 1

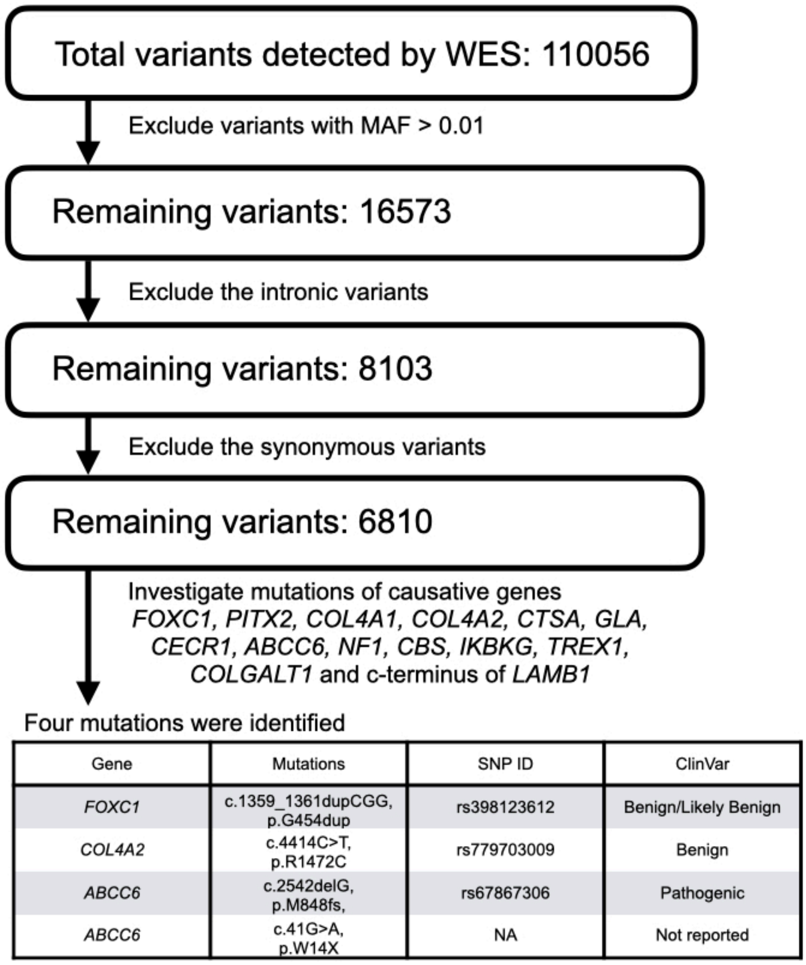

Gene	Mutations	SNP ID	ClinVar
FOXC1	c.1359_1361dupCGG, p.G454dup	rs398123612	Benign/Likely Benign
COL4A2	c.4414C>T, p.R1472C	rs779703009	Benign
ABCC6	c.2542delG, p.M848fs,	rs67867306	Pathogenic
ABCC6	c.41G>A, p.W14X	NA	Not reported

Supplementary Figure 1. Workflow of identifying causative mutations detected by WES

First, we excluded detected variants with a MAF > 0.01. Variants with intronic and synonymous variants were excluded from the remaining variants. We investigated mutations in the causative genes among the remaining variants. Finally, we evaluated the pathogenicity of each mutation using the ClinVar website or previous reports. This figure shows the results of WES for the patient with PXE. We identified four mutations among the causative genes of CSVD. Two of these were benign or likely benign in ClinVar. p.M848fs mutation in *ABCC6* is the most frequent causative mutations of PXE in Japan.[4]

Supplementary figure 2

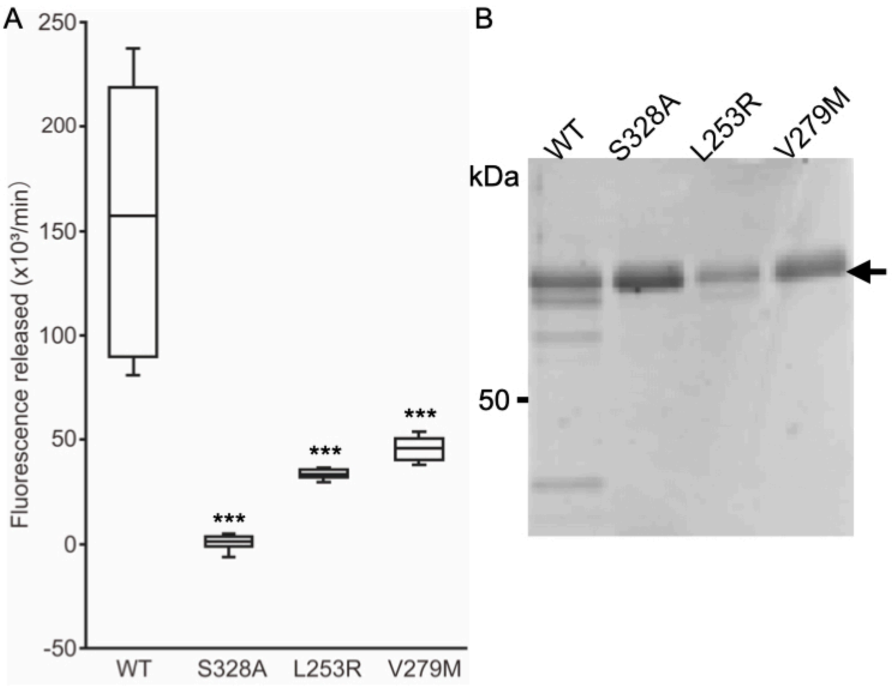

Supplementary Figure 2. Protease activity of each mutant HTRA1 protein

(A) Protease activities of missense HTRA1s identified in this study. Activities were calculated from the slope of the linear portion of normalized fluorescence vs. time plots. The boxplots show the values from two independent experiments of three samples of each protein. WT and S328A indicate the positive and negative controls, respectively. Protease activities were statistically compared between the WT and the other three HTRA1s with an ANOVA followed by the Bonferroni correction. ***p-value < 0.0001 for protease activities of each HTRA1 relative to WT.

Supplemental material BMJ Publishing Group Limited (BMJ) disclaims all liability and responsibility arising from any reliance placed on this supplemental material which has been supplied by the author(s)

(B) SDS-PAGE of WT and missense mutant HTRA1 proteins used in the protease assay. Black arrows indicate the full-length band of HTRA1 tagged with myc-His6.

Supplementary figure 3

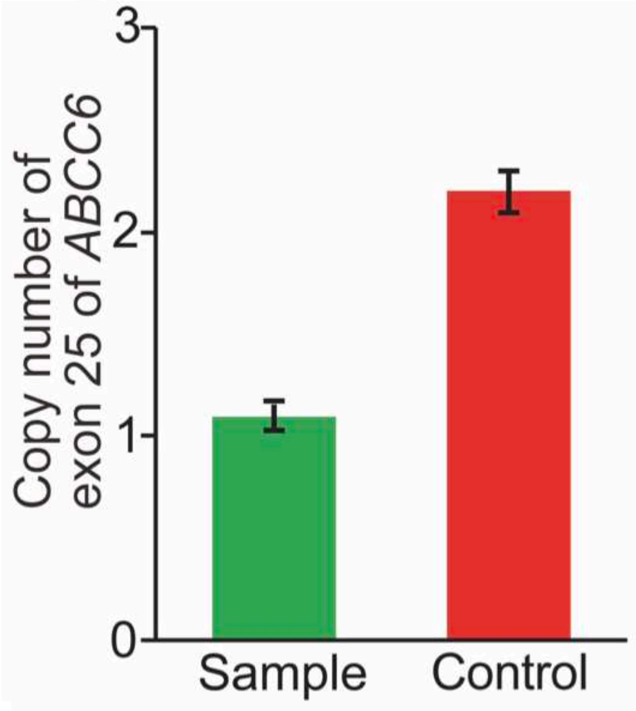

Supplementary Figure 3. ***ABCC6*** **exon 25 CNVs**

The copy number of *ABCC6* exon 25 is shown as a bar graph. The vertical axis indicates the copy number of exon 25 in *ABCC6*. Green and red bars indicate the copy number of exon 25 of *ABCC6* in a patient with decreased copy numbers of 7–10, 16–17, and 21–25 of *ABCC6* and controls. Mean values from three samples for each ddPCR are shown. I-bars indicate the standard error (SE).
